# Differential effects of antiretroviral treatment on immunity and gut microbiome composition in people living with HIV in rural versus urban Zimbabwe

**DOI:** 10.1186/s40168-023-01718-4

**Published:** 2024-02-03

**Authors:** Angela Sofia Burkhart Colorado, Alessandro Lazzaro, Charles Preston Neff, Nichole Nusbacher, Kathryn Boyd, Suzanne Fiorillo, Casey Martin, Janet C. Siebert, Thomas B. Campbell, Margaret Borok, Brent E. Palmer, Catherine Lozupone

**Affiliations:** 1 Department of Biomedical Informatics, University of Colorado Anschutz Medical Campus, Aurora, CO 80045 USA; 2 Department of Public Health and Infectious Diseases, Sapienza University of Rome, 00185 Rome, Italy; 3 Department Medicine, University of Colorado Anschutz Medical Campus, Aurora, CO 80045 USA; 4 Department of Clinical Research, London School of Hygiene and Tropical Medicine, Keppel St, London, WC1E 7HT England; 5 Faculty of Medicine and Health Sciences, University of Zimbabwe, Harare, Zimbabwe

**Keywords:** HIV, Intestinal microbiome, ART response, Immune activation and exhaustion, Inflammation, Urban, Rural

## Abstract

**Background:**

The widespread availability of antiretroviral therapy (ART) has dramatically reduced mortality and improved life expectancy for people living with HIV (PLWH). However, even with HIV-1 suppression, chronic immune activation and elevated inflammation persist and have been linked to a pro-inflammatory gut microbiome composition and compromised intestinal barrier integrity. PLWH in urban versus rural areas of sub-Saharan Africa experience differences in environmental factors that may impact the gut microbiome and immune system, in response to ART, yet this has not previously been investigated in these groups. To address this, we measured T cell activation/exhaustion/trafficking markers, plasma inflammatory markers, and fecal microbiome composition in PLWH and healthy participants recruited from an urban clinic in the city of Harare, Zimbabwe, and a district hospital that services surrounding rural villages. PLWH were either ART naïve at baseline and sampled again after 24 weeks of first-line ART and the antibiotic cotrimoxazole or were ART-experienced at both timepoints.

**Results:**

Although expected reductions in the inflammatory marker IL-6, T-cell activation, and exhaustion were observed with ART-induced viral suppression, these changes were much more pronounced in the urban versus the rural area. Gut microbiome composition was the most highly altered from healthy controls in ART experienced PLWH, and characterized by both reduced alpha diversity and altered composition. However, gut microbiome composition showed a pronounced relationship with T cell activation and exhaustion in ART-naïve PLWH, suggesting a particularly significant role for the gut microbiome in disease progression in uncontrolled infection. Elevated immune exhaustion after 24 weeks of ART did correlate with both living in the rural location and a more Prevotella-rich/Bacteroides-poor microbiome type, suggesting a potential role for rural-associated microbiome differences or their co-variates in the muted improvements in immune exhaustion in the rural area.

**Conclusion:**

Successful ART was less effective at reducing gut microbiome-associated inflammation and T cell activation in PLWH in rural versus urban Zimbabwe, suggesting that individuals on ART in rural areas of Zimbabwe may be more vulnerable to co-morbidity related to sustained immune dysfunction in treated infection.

Video Abstract

**Supplementary Information:**

The online version contains supplementary material available at 10.1186/s40168-023-01718-4.

## Introduction 

Human immunodeficiency virus 1 (HIV-1) infection is characterized by progressive infection and depletion of CD4 + T cells, chronic immune activation, and immune exhaustion that predisposes the infected individual to opportunistic infections and cancers defining acquired immunodeficiency syndrome (AIDS). Antiretroviral therapy (ART) has dramatically improved health outcomes in PLWH. However, ART coverage remains suboptimal in many parts of the developing world, including in sub-Saharan Africa (SSA) where about 70% of the global HIV epidemic is concentrated [1], and is particularly challenging in rural areas [2]. Even with successful ART, PLWH often have chronic immune activation and elevated inflammation [3] that has been linked with poor CD4 + T cell recovery [4] and the premature onset of HIV-related non-AIDS-defining comorbidities [5]. Understanding factors related to chronic immune activation with ART is essential for devising strategies to protect the health of PLWH.

Factors that can drive chronic immune activation in PLWH on effective ART include co-infections, persistent antigen stimulation from residual viremia and the intestinal microbiome [5]. This complex community of microbes has been of intense interest in HIV-infected populations, in part because HIV-1 disrupts gut associated lymphoid tissue (GALT) causing gut mucosa damage that allows for the translocation of inflammatory bacterial components, which is strongly tied to disease progression [6]. Studies conducted in the USA and Europe have found gut microbiome differences in HIV-infected populations that could be a result of HIV-driven immune dysfunction, ART drugs, or lifestyle factors [7–9]. These altered microbiomes have been shown to correlate with chronic T cell activation in vivo and to drive higher T cell activation in vitro [10] and cytokine production ex vivo [11]. Relatively few studies have investigated effects of HIV-1 or ART on the gut microbiome in SSA [12–15], and yet knowledge from the developed world may not be generalizable for several reasons. First, there are dramatic differences in gut microbiome composition in healthy individuals in the developing versus the developed world that are associated with differences in diet and environmental factors [16]. Second, the predominant mode of HIV-1 transmission differs in SSA compared to other regions, leading to related differences in demographic factors and behaviors that can influence gut microbiome composition [17]. Finally, concomitant use of the antibiotic cotrimoxazole to prevent opportunistic infections with ART regardless of CD4 + T cell count is more common in SSA compared to developed countries [18].

Individuals living near urban centers versus in rural areas of SSA have differences in environmental exposures such as water source or diet that may impact their gut microbiome composition and immune response to microbes [19, 20]. There may also be different responses to ART in rural areas, although studies generally have focused on measuring rates of treatment failure rather than on differences in immune response following virologic control [2, 21]. To gain a further understanding of effects of HIV treatment on the gut microbiome and immune activation and inflammation in PLWH in SSA, and whether living in a rural versus urban area can influence the effects ART, we designed a prospective longitudinal observational study of participants from rural and urban hospitals in Zimbabwe. We examined T cell activation, exhaustion and trafficking markers, and inflammatory plasma cytokines and gut microbiome composition among ART-naïve and ART-experienced PLWH and healthy controls and assessed effects of 24 weeks of ART and cotrimoxazole using longitudinal analysis.

## Results

### Demographic and clinical characteristics of study cohort

We recruited 162 individuals with approximately equal numbers from the urban Mabvuku Polyclinic in the city of Harare and the Mutoko District Hospital, located in a district of around 161,000 people [22] 146 km from Harare that services surrounding rural villages. Blood-immune profile and fecal microbiome composition were evaluated in samples collected at two timepoints 24 weeks apart in (1) PLWH who were not on ART at the first timepoint but who subsequently commenced first-line ART with efavirenz/lamivudine/tenofovir disoproxil fumarate (EFV/3TC/TDF) and the prophylactic antibiotic cotrimoxazole (ART Naïve); (2) PLWH who were on this same ART regimen and cotrimoxazole at both timepoints (ART experienced); and (3) people without HIV, hereafter referred to as healthy controls (HC). Of the 162 enrolled individuals, 14 from the ART naïve cohort were excluded because they had HIV viral load below 20 copies/mL at the baseline visit, which is inconsistent with their declared treatment status (Figure S1). Because we wanted to evaluate the effects of successful ART, we also excluded 6 individuals in the ART experienced cohort who had a viral load > 200 copies/mL at baseline (Figure S1). Baseline values of 142 individuals were analyzed with 67 individuals in the ART naïve cohort, 33 in the ART experienced cohort, and 42 HC (Table 1). Furthermore, for longitudinal (intra-cohort) and microbiome analysis, additional individuals were excluded because they either (1) were lost to follow-up at the 24 week visit (*n* = 14), or (2) were in the ART-naïve cohort and did not have a viral load < 200 at the second timepoint (*n* = 15), indicating a lack of response to ART. Longitudinal (intra-cohort) analyses thus included 226 samples from 113 total individuals (Figure S1).

**Table 1:** Summary of demographics

	ART Naïve PLWH (Naïve)	ART Experienced PLWH (Exp)	Healthy Controls (HC)	p-value		
	(N=67)	(N=33)	(N=42)	Naïve vs Exp	Naïve vs HC	Exp vs HC
**Arm**				0.84	0.87	0.74
Rural	34 (50.7%)	16 (48.5%)	22 (52.4%)			
Urban	33 (49.3%)	17 (51.5%)	20 (47.6%)			
**Sex**				0.53	0.73	0.79
Female	39 (58.2%)	17 (51.5%)	23 (54.8%)			
Male	28 (41.8%)	16 (48.5%)	19 (45.2%)			
**Age (years)**	35 [19, 51]	46 [19, 64]	35.5 [18, 67]	<0.0001	0.69	<0.0001
**BMI (kg/m^2)**	22.7 [15.8, 33.4]	20.2 [14.6, 27.8]	22.6 [16.9, 35]	0.018	0.32	0.004
**BMI Category**				0.057	0.22	0.014
Severe Thinness <16.0	1 (1.5%)	2 (6.1%)	0 (0%)			
Moderate Thinness <17.0	0 (0%)	0 (0%)	1 (2.4%)			
Mild Thinness <18.5	3 (4.5%)	5 (15.2%)	1 (2.4%)			
Normal 18.5–24.9	51 (76.1%)	22 (66.7%)	28 (66.7%)			
Overweight ≥25.0	10 (14.9%)	4 (12.1%)	10 (23.8%)			
Obese ≥30.0	2 (3.0%)	0 (0%)	2 (4.8%)			
**CD4 T cells count (cells/μL)**	257 [10, 824]	412 [77, 838]	NA [NA, NA]	0.001	NA	NA
**CD4 T cells (%)**	22.2 [0, 48.8]	36.7 [0.1, 55.7]	55.8 [27.8, 70.4]	0.00037	<0.0001	<0.0001
**CD4/CD8 T cell ratio**	0.3 [0, 1.3]	0.7 [0, 1.5]	1.7 [0.4, 3.3]	0.00069	<0.0001	<0.0001
**ART exposure (years)**	NA [NA, NA]	7 [1.8, 21.7]	0 [0, 0]	NA	NA	NA
**Co-trimoxazole exposure (months)**	0 [0, 7]	84 [0, 138]	0 [0, 0]	<0.0001	NA	NA
**Viral Load (copies/mL)**	26491 [36, 1159529]	0 [0, 103]	NA [NA, NA]	<0.0001	NA	NA
**Both Visit**				0.2	0.88	0.27
No	7 (10.4%)	1 (3.0%)	4 (9.5%)			
Yes	60 (89.6%)	32 (97.0%)	38 (90.5%)			

Clinical and demographic characteristics of the study population by cohort at the baseline visit. *p*-values were calculated using the Mann-Whitney *U* test. BMI categories were determined using the World Health Organization (WHO) standards [23]. NA represents values not collected/relevant to a particular cohort. Values are reported as the median with the minimum and maximum range in brackets

Cohorts had similar proportions of females versus males. ART-naïve PLWH and HC had a similar median age of 35 and 35.5 respectively while ART-experienced PLWH were significantly older (median age of 46) (Table 1). Median body mass index (BMI) was in the normal range for the overall population (BMI = 22) and ART-experienced PLWH had lower BMI compared to both ART-naïve PLWH and HC (Table 1); this pattern was significant in males and not females in stratified analysis (Table S1) and women had a significantly higher BMI than men. Notably, 43.8% of ART-experienced men and 0% of ART-experienced women were in the underweight categories (Table S1). The median duration of ART and cotrimoxazole treatment among the ART experienced cohort at baseline was 7 years. The median duration of cotrimoxazole treatment at baseline was 0 months in the ART naïve cohort (Table 1), but significantly longer in the rural (median of 0.2 months) compared to the urban (median of 0 months) location (Table S2).

### Cross-sectional and longitudinal analysis of immune phenotype

As expected, the ART-naïve cohort had higher HIV viral loads and lower CD4 + T cell count, CD4 + T cell percent, and CD4/CD8 T cell ratio compared to the ART-experienced cohort at baseline (Table 1; Figure S2). CD4 + T cell percent and CD4/CD8 T cell ratio were not significantly different at baseline between the urban and rural location in ART-naïve or experienced PLWH, but were higher in HC in the rural area (Table S2). CD4 + T cell count, CD4 + T cell percent, and CD4/CD8 T cell ratio were higher in ART-naïve and ART-experienced women compared to men (Table S1). All but 15 (22.4%) of the ART naïve individuals reached virologic control, defined as viral load < 200 copies of HIV-1/mL of plasma, after 24 weeks of therapy and 6 (15.4%) of the 39 individuals who were originally recruited into the ART experienced cohort had uncontrolled HIV infection at baseline despite ART (Figure S1). Levels of virologic control with ART were not significantly different between the urban and rural sites. The individuals that remained viremic with treatment were removed from longitudinal analyses (Figure S1). A significant increase in CD4 + T cell percent and CD4/CD8 T cell ratio was observed after 24 weeks of treatment among individuals in the ART-naïve cohort who reached virologic control in both the urban and rural sites (Figure S2).

We next used flow cytometry to gain a deeper understanding of the effects of successful ART on CD4 + and CD8 + T-cell populations in blood. The immune characterization included the following: (1) *chronic immune activation* using HLA-DR and CD38 as markers, a common measure of disease progression in studies of HIV [24–29]; (2) a marker of *immune exhaustion*, PD-1, which prior studies have shown to increase in PLWH [30–32]; and (3) *mucosal trafficking* by examination of CD103 expression, which can either increase in blood with challenge as populations expand, or decrease as they traffic from blood to mucosal sites [33, 34]. We also evaluated *inflammation*, by measuring plasma levels IL-6 and CRP using ELISA, both of which have been shown to increase in PLWH [35, 36].

#### Immune activation, exhaustion, and inflammation

**Fig. 1:**
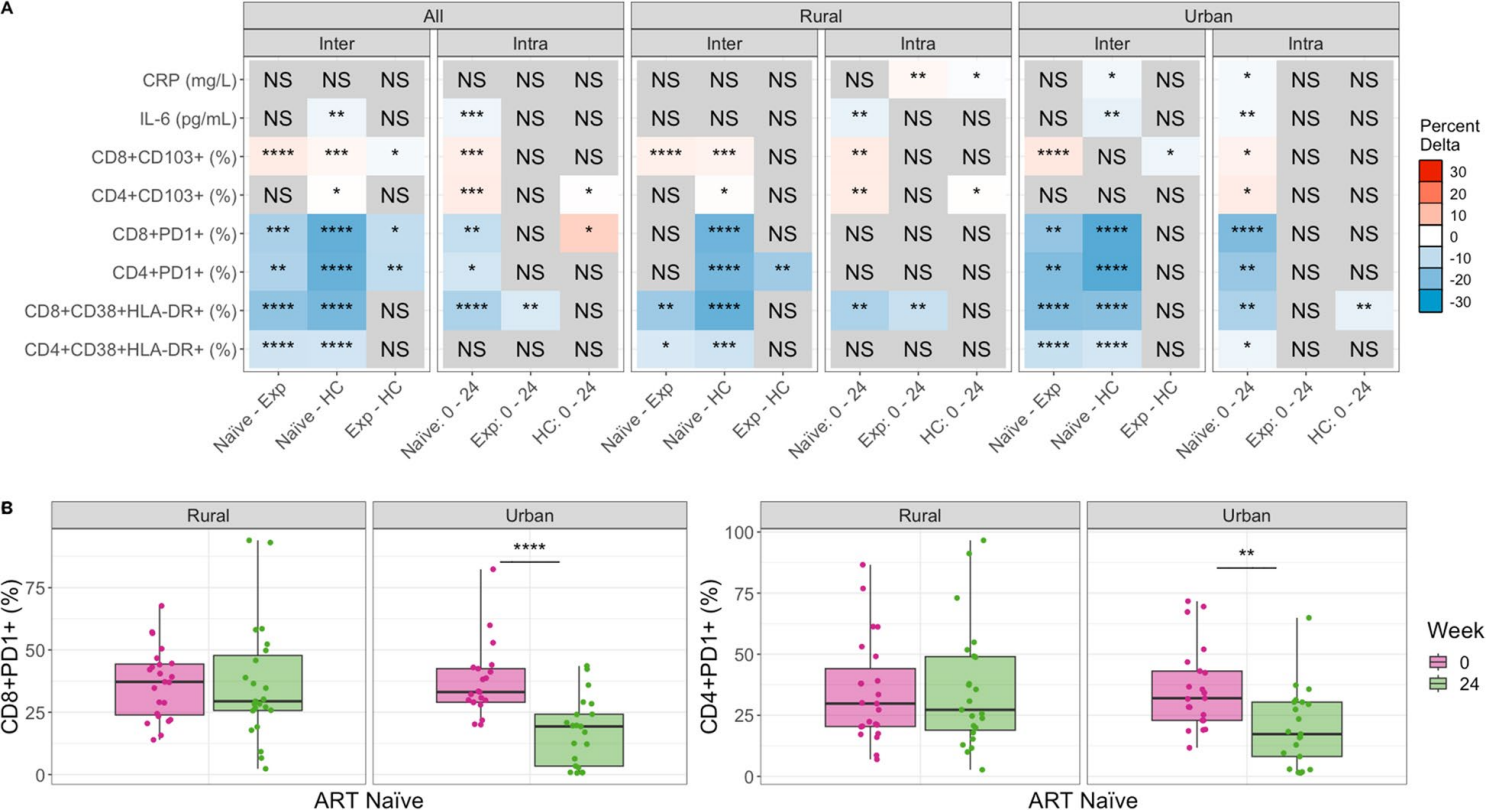
Differences in immune markers across cohorts and over time, before and after commencement of ART. Comparing immune marker values between cohorts (inter) and across timepoints (intra). Inter-cohort comparisons were performed on baseline (week 0) samples using a Kruskal–Wallis with a Dunn’s post hoc test. Intra-cohort comparisons compared week 0 to 24 using a paired Mann–Whitney *U* test. Significant relationships are colored by the mean change in value to show directionality: For any given comparison (e.g., naïve – Exp), the box is colored red (up) if the average value of Exp is higher than that of naïve. If blue (down), the average value of Exp is lower than naïve. The intensity of the color indicates the strength of the change. Detailed plots for significant relationships are shown in Figure S3 (CD8 + CD38 + HLA-DR + and CD4 + CD38 + HLA-DR +), Figure S4 (CD4 + PD1 + and CD8 + PD1 +), Figure S5 (IL-6 and CRP), and Figure S6 (CD8 + CD103 + and CD4 + CD103 +). **B** Stratified analysis of CD8 and CD4 PD1 percent change over time by location. *p*-values are coded as “****” between (0, 0.0001), “***” (0.0001, 0.001), “**” (0.001, 0.01), and “*” (0.01, 0.05), with square brackets indicating that the endpoints are included in the interval. NS not significant, Naïve ART-naïve cohort, Exp ART-experienced cohort

As expected, based on prior studies [30, 32, 34, 35], ART-naïve PLWH had higher levels of chronically activated (CD38 + HLA-DR +) and exhausted (PD1 +) CD4 + and CD8 + T cells compared to HC (Fig. 1, Figures S3, S4). Compared to ART-naïve, PLWH who were ART-experienced at baseline had significantly lower levels of chronically activated and exhausted CD4 + and CD8 + T cells (Fig. 1, Figures S3, S4), showing expected improvement with ART. However, when conducting the same analyses stratified by the urban versus rural location, significantly lower T cell exhaustion levels in ART-experienced versus ART-naive PLWH were found only in the urban and not the rural location (Fig. 1, Figures S3, S4). ART-experienced PLWH also had significantly higher levels of CD4 + and CD8 + T cell exhaustion compared to HC, indicating incomplete recovery with ART, but statistically significant differences in CD8 + T cell exhaustion were observed in the rural and not urban cohort in stratified analysis (Fig. 1, Figure S4). The inflammatory marker IL-6 also showed the highest level at baseline in the ART naïve cohort, followed by the ART experienced cohort, while the HC had the lowest levels. However, only a reduction in IL-6 between ART-naïve PLWH and the HC was statistically significant, and this difference was significant in the urban and not rural location in stratified analyses (Fig. 1A, Figure S5). Taken together, these results show ART-experienced individuals to have expected improvements in inflammation and T cell exhaustion compared to ART-naïve PLWH in the urban but not the rural area. Accordingly, ART-experienced individuals in the rural and not the urban area had significant differences from HC in exhaustion phenotypes.

Longitudinal analysis of the ART naïve cohort showed consistent results. There was a significant reduction in CD4 + and CD8 + T cell exhaustion and CD8 + but not CD4 + chronic T cell activation with 24 weeks treatment overall (Fig. 1, Figures S3, S4). However, again this was driven by improvements only in the urban area; individuals from the urban area showed a significant reduction with 24 weeks of ART in all CD4 + and CD8 + T cell activation and exhaustion markers but the PLWH in the rural area showed a significant reduction in CD8 + T cell activation only (Fig. 1). ART-naïve PLWH in the urban area had a significant reduction in both IL-6 and CRP following 24 weeks of ART, but in the rural area only IL-6 was reduced. Overall, these results suggest that the people living in the urban location exhibit better improvement in T cell activation and exhaustion levels and inflammation with ART. This is the case even though we restricted our analyses to include only individuals with controlled viral replication and the rural and urban sites did not differ in the percent of individuals who achieved virologic control with ART.

One result suggesting a potential confounder is that the HC exhibited increased CD8 + T cell exhaustion over time, even though no intervention occurred in this cohort. Similarly, the ART-experienced cohort showed an increase in CRP over time in the rural and not the urban location (Fig. 1A). Furthermore, CD8 + CD38 + HLA-DR + levels decreased over time in HC in the urban and not the rural area. Changes over time without an intervention may be related to increased socio-economic stressors in Zimbabwe between January 2018 and August 2019 [37], when these samples were collected. To correct for these potential confounders, we also applied a linear modeling approach to determine whether the changes observed with treatment of the ART-naïve cohort were greater than those observed over time in the HC cohort (see Methods Model M1; Figure S7). These results suggested that improvements in CD4 + PD1 + and CD8 + PD1 + T cells with 24 weeks of ART may have been underestimated and improvements in CD8 + CD38 + HLA-DR + T cells with ART in the urban area may be overestimated. They also showed that CRP results were impacted by confounding effects over time. When measured as change in the ART-naïve cohort relative to the HC, we found that CRP decreased with 24 weeks of ART in the urban area, as would be expected, but actually increased in the rural area (Figure S5).

#### Mucosal trafficking

One goal of this study was to relate immune markers in HIV to the intestinal microbiome. Thus, we also evaluated T cells expressing the mucosal trafficking marker CD103, since some of these cells might be trafficking to or from intestinal sites. CD8 + CD103 + T cells were highest in the ART-experienced cohort and lowest in the ART-naïve. CD4 + CD103 + T cells were significantly higher in HC compared to ART-naïve at baseline (Fig. 1, Figure S6). Change over time in the ART naïve cohort showed that both CD4 + and CD8 + T cells expressing CD103 increased with treatment (Fig. 1A). The HC cohort showed a significant decrease in CD4 + CD103 + T cells over time in the rural area (Fig. 1A), so linear modeling showed that the increase over time in ART-naïve PLWH relative to HC was more pronounced (Figure S6).

### Cross-sectional and longitudinal analysis of microbiome diversity

#### Alpha diversity

**Fig. 2:**
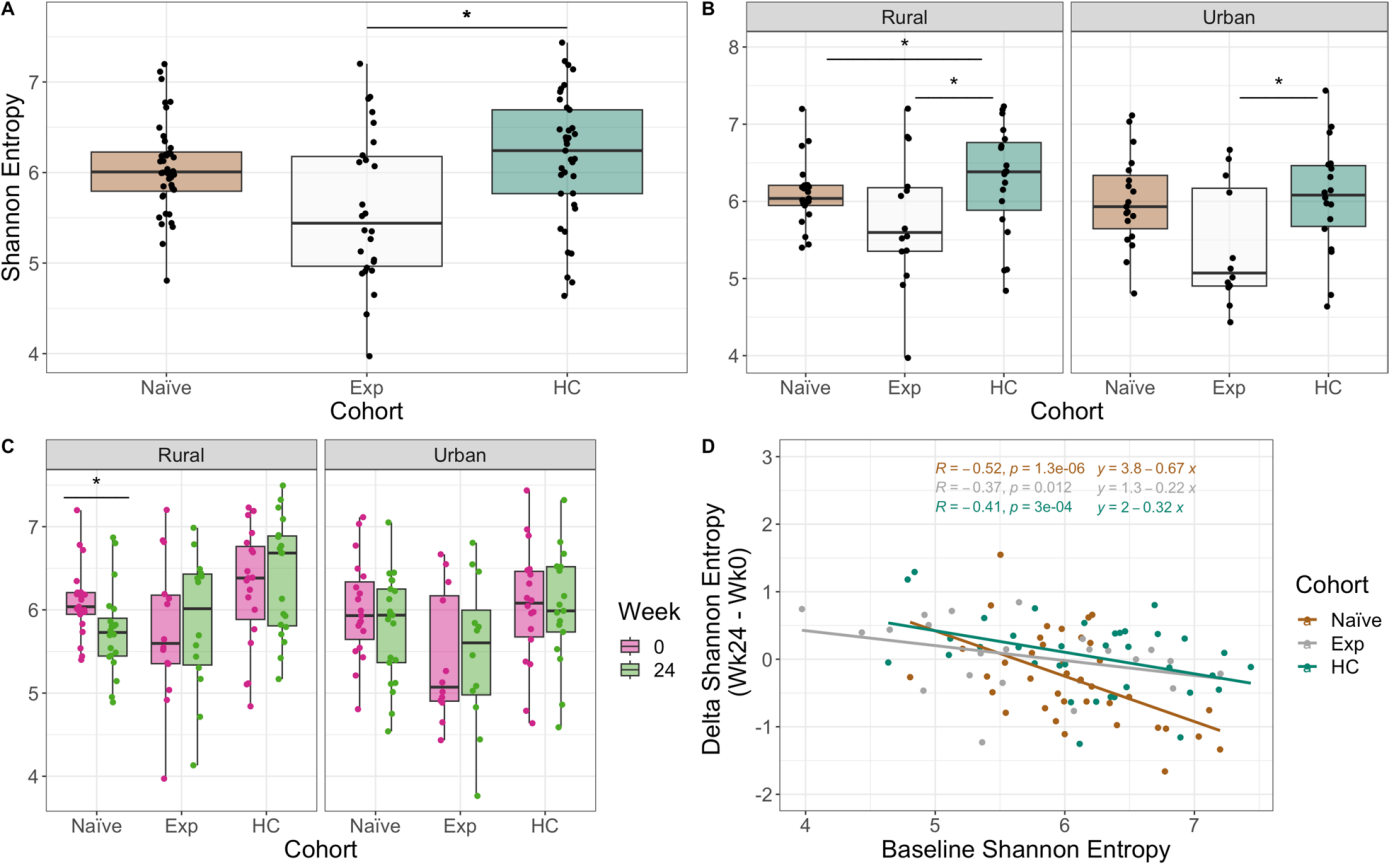
Alpha diversity across cohorts stratified by time and location. Shannon entropy differences between cohorts at baseline. *p*-values were calculated using a Kruskal–Wallis test with a Dunn’s post hoc (pairwise comparisons shown).** B** Same as **A** stratified by location. **C** Shannon entropy between timepoints for each cohort stratified by location. *p*-values were calculated using a paired Mann–Whitney *U* test. **D** Linear regression of the difference in Shannon entropy (Shannon entropy at week 24 minus week 0) by baseline Shannon entropy (Shannon entropy at week 0). *p*-values are coded as “****” between (0, 0.0001), “***” (0.0001, 0.001), “**” (0.001, 0.01), “*” (0.01, 0.05), with square brackets indicating that the endpoints are included in the interval

We next evaluated intestinal microbiome composition using 16S ribosomal RNA (rRNA)-targeted sequencing of fecal samples. One potential confounder in understanding the effects of ART on the microbiome in PLWH is the concomitant use of the antibiotic cotrimoxazole. There was also some exposure of ART-naïve individuals to cotrimoxazole at baseline, and this was greater in people living in the rural versus urban location (Table 1). Despite this, ART-naïve individuals in the rural area did not have significantly lower alpha diversity (measured with Shannon entropy [38]) at baseline compared to urban (Mann–Whitney *U* test, *p* > 0.05) and length of cotrimoxazole treatment at baseline did not correlate with alpha diversity (Figure S8). Individuals in the ART naïve cohort had reduced Shannon entropy compared to HC in the rural and not the urban location (Fig. 2A).

The ART-naïve cohort had the lowest alpha diversity at baseline in both locations (Fig. 2A,B). ART-naïve patients had a significant decrease in alpha diversity following 24 weeks of ART in the rural location only (Fig. 2C). There are two distinct processes that may underlie a relationship between treatment and microbiome alpha diversity: (1) the ART drugs or cotrimoxazole might decrease diversity through direct effects on the microbiome or (2) immune or gut barrier function improvements that occur with viral control could restore diversity of a compromised microbiome. We thus hypothesized that increasing or decreasing diversity with ART might depend on baseline values. To test this hypothesis, we used linear regression to evaluate whether the change in Shannon Entropy following 24 weeks of treatment was significantly related to baseline Shannon Entropy values (Fig. 2D, see Methods Model M2). Indeed, individuals with the highest baseline diversity showed the greatest loss in diversity, indicating a greater potential importance of direct drug effects. Alternately, those with the lowest baseline diversity showed a positive change in alpha diversity on average in both urban and rural areas, indicating that ART can potentially restore diversity in those with higher levels of HIV-driven microbiome disturbance.

#### Beta diversity

**Fig. 3:**
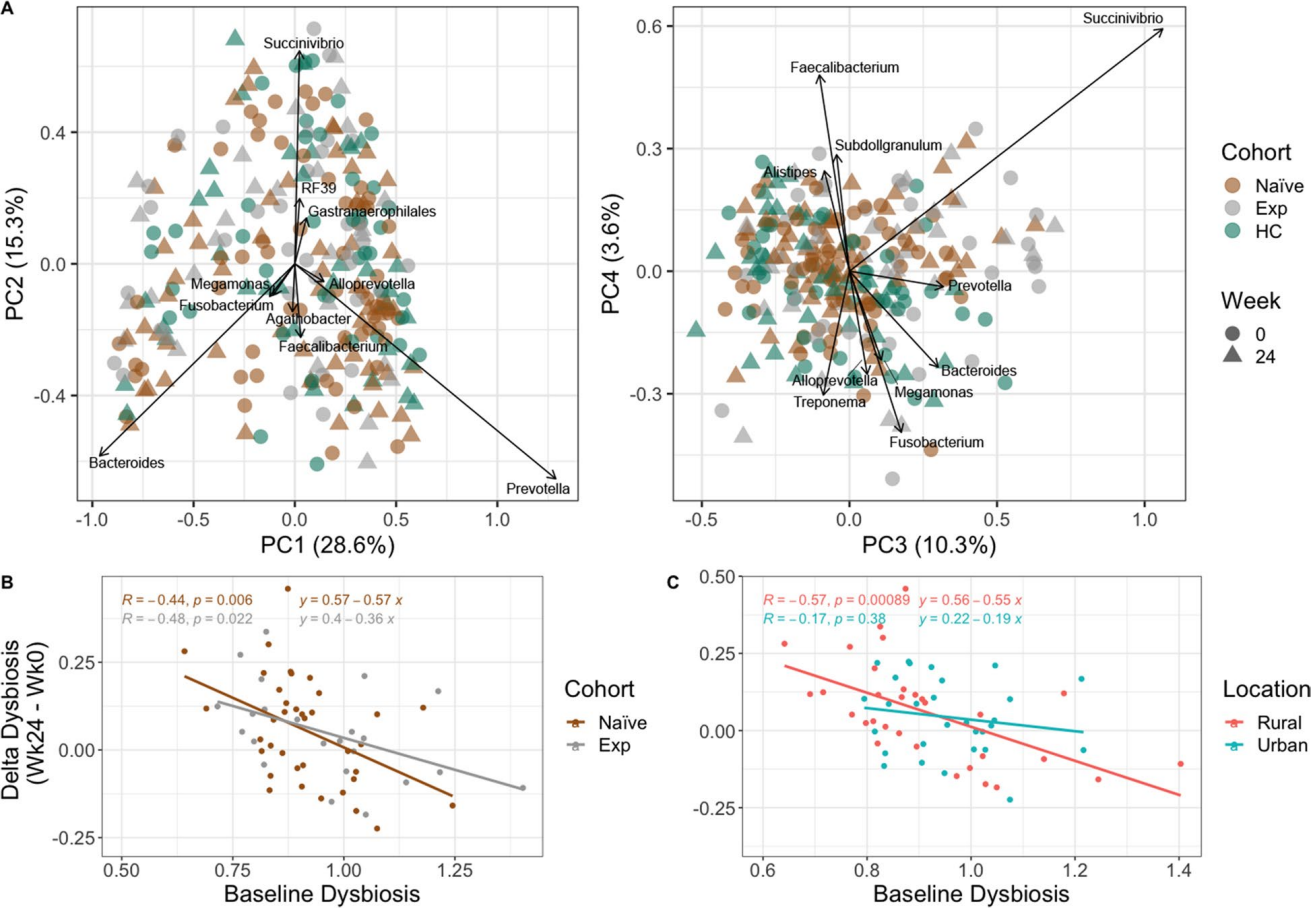
Biplots of weighted UniFrac PCoA axes and delta dysbiosis analyses stratified by cohort and location. **A** Plotted weighted UniFrac PCoA axes that account for over 50% of the variance with important genera (importance defined as those farthest from the origin using Euclidean distance) indicated. **B** Linear regression model showing delta dysbiosis as a function of baseline dysbiosis stratified by cohort. Dysbiosis for each sample in ART-naïve and experienced cohorts was calculated by taking the average weighted UniFrac distance from each sample to all healthy controls (HC). This average was calculated on values of the same timepoint. For example, if the sample had been taken at week 0, the dysbiosis score (average distance to HC) was calculated with values only at week 0. Delta dysbiosis was calculated by taking the difference in dysbiosis between week 24 and 0. **C** Same analysis as **B** but stratified by location

Individuals in Zimbabwe had a microbiome composition typical of those previously described in studies of SSA [39], with relatively high relative abundance of bacteria in the genus *Prevotella* (17.71% mean relative abundance ± 13.07%) and low *Bacteroides* (9.42% mean relative abundance ± 9.26%) (Figure S9). In a principal coordinate analysis (PCoA) of weighted UniFrac [40] values to visualize beta diversity, principal coordinate 1 (PC1) separated individuals by differences in *Prevotella* and *Bacteroides* genera (Fig. 3A). We used a linear model to explore relationships between PC1–PC4 and HIV/ART status, rural versus urban location, BMI, cotrimoxazole, CD4 + percent, CD4/CD8 ratio, viral load, and Shannon entropy (see Methods Model 3; Table S3). We found that higher levels of PC1 were found in those in the rural location, which is consistent with prior studies showing more *Prevotella*-rich microbiomes in rural versus urban areas in SSA [20]. PC2 correlated positively with Shannon entropy, BMI, and length on cotrimoxazole. PC3 correlated negatively with Shannon entropy and length on cotrimoxazole, and PC4 correlated positively with ART status (Table S3; Fig. 3B). Treatment (*p*-value = 0.039) but not HIV-infection status (*p* = 0.111) had a significant effect on beta diversity based on Adonis (see Methods Model M4) and living in the rural versus urban location also had a significant effect on beta diversity (Adonis, *p* = 0.035; see Methods Model M5). PCoA plots stratified by location and week are shown in Figure S10.

We also used beta diversity measures to estimate dysbiosis as the average weighted UniFrac distance between PLWH and HC (Fig. 3B, C, Figure S11). ART experienced PLWH showed significantly higher dysbiosis compared to ART-naïve PLWH in the urban and not rural area (Figure S11), with the rural area actually showing the opposite trend. However, individuals in the rural area had higher exposure to cotrimoxazole at baseline, and the time of cotrimoxazole at baseline did correlate significantly with dysbiosis (Figure S8), which may in part explain why change was only observed in the urban cohort. No significant change in dysbiosis over time was found in ART-naïve and experienced cohorts, including when stratifying for location (Figure S10). We hypothesized that a change in dysbiosis with ART could manifest as increased dysbiosis due to direct drug effects, or decreased dysbiosis due to ART driven immunologic improvements, and thus may be influenced by baseline dysbiosis. Indeed, change in dysbiosis was significantly related to baseline values in a linear regression (see Methods Model M6), particularly in the rural location (Fig. 3C), with those with the highest dysbiosis at baseline having improvement (negative delta) following 24 weeks of ART/cotrimoxazole treatment and individuals with the lowest dysbiosis at baseline showing worsening dysbiosis (positive delta) at week 24. This same pattern was also statistically significant in the ART-experienced cohort though with a smaller effect size (slope) (Fig. 3B), suggesting continuous effects of ART on dysbiosis over time.

#### Differential abundance analysis

To understand which genera significantly differed between cohorts, we used ANCOM-BC2 [41, 42], a differential abundance (DA) analysis package for compositional microbiome data that allows for regression modeling to control for potential confounding factors. Specifically, we determined which genera differed between HC and the ART-experienced or ART-naïve cohorts in models that also included the effect of time, viral load, and location to control for confounders. We used both baseline values only (see Methods Model M7) so that we could compare the naïve cohort before ART treatment to the other two cohorts, and a mixed effects model analysis with data from both timepoints (see Methods Model M8), although in the ART-naïve versus HC comparison, it is important to note that this includes individuals both before and after ART, and so results will also in part be driven by ART. Analyses were done on bacterial genera classified with the Silva taxonomy (version 138) [43].

**Fig. 4:**
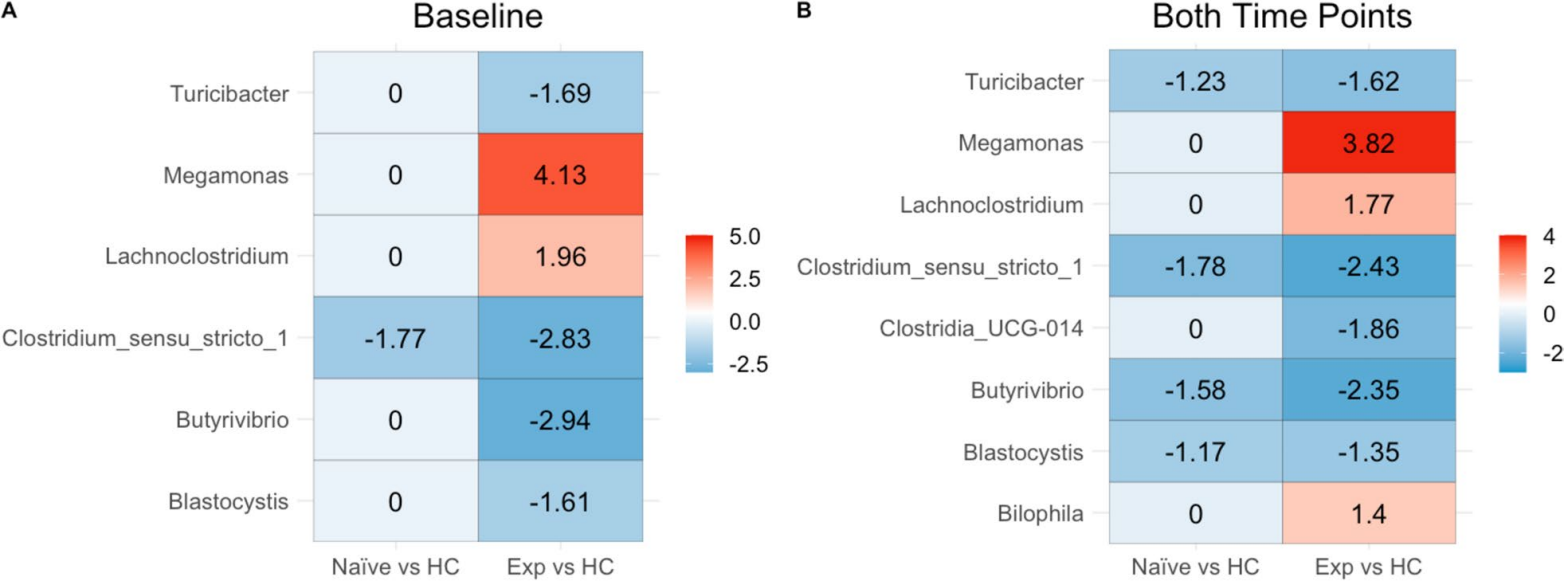
Differential abundance of genera relative to healthy controls. **A** Using ANCOM-BC, significant differential log-fold changes between microbes were calculated relative to healthy controls using only baseline values. The model used also evaluated the effect of location and viral outcome. A zero means that there is no significant difference in the genus between cohorts. **B** Similar to **A**, however both timepoints were included in the model; therefore, time was added to the model, and we controlled for dependence between samples belonging to the same patient

The ART-naïve cohort showed only a significant decrease in the *Clostridium_sensu_stricto_1* genus when compared to HC at baseline. With two timepoints (so also including samples from after 24 weeks of ART), the ART-naïve versus HC comparison additionally showed decreases in *Turicibacter*, *Blastocystis*, and *Butyrivibrio* (Fig. 4B). Neither location nor viral load was found to impact DA at baseline.

Comparisons between ART experienced PLWH and HC baseline values showed an increase in *Lachnoclostridium* and *Megamonas* and a decrease in *Turicibacter*, *Butyrivibrio*, *Blastocystis*, and *Clostridium_sensu_stricto_1* (Fig. 4A). In models created using samples from both timepoints, a decrease in *Clostridia_UCG-014* in the ART experienced and an increase in *Bilophila* was additionally detected (Fig. 4B). Finally, we also evaluated change over time in the ART-naïve cohort (see Methods Model M9) to detect longitudinal microbiome changes with 24 weeks of ART using mixed linear model. Only genus *Lachnoclostridium* was significant and decreased over time (*p*-value 7.75e-05).

### Integrative analysis of immune markers and microbiome

**Fig. 5:**
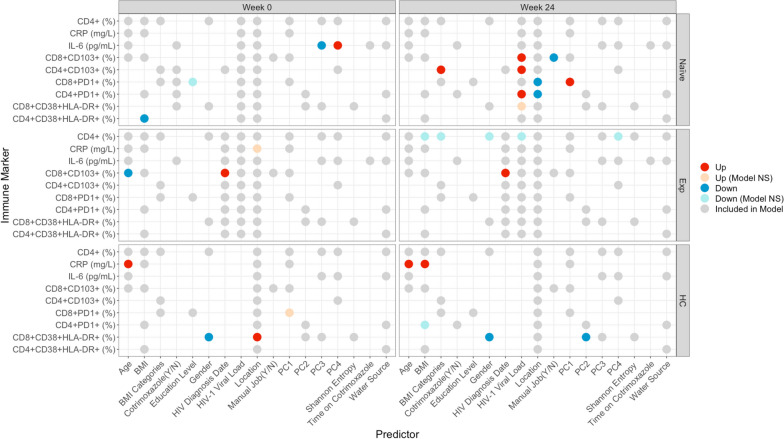
Predictive models for immune markers. Each row within each square represents one model. Circles represent predictors included in models. Predictors were determined by backwards stepwise regression feature selection. Location was retained in all models, viral load in the 2 PLWH cohorts, and HIV diagnosis date in the ART-experienced cohort. Predictors that had a significant impact on the model are colored red (↑/positive) or blue (↓/negative). Gender (up) represents higher values in males; location (up) represents higher values in the rural location; BMI categories (up) represent higher values with higher BMI; water source (up) represents higher values in people who drink from wells as opposed to tap; education level (up) represents higher values in people who went to secondary school as compared to tertiary; manual job (up) represents higher values in those who do work manual jobs as compared to those who do not. “Model NS” means that the overall model was not significant despite the predictor being significant. Gray circles represent non-significant predictors

To gain further insight into relationships between gut microbiome composition and immune phenotypes, we used linear models with the immune markers described in Fig. 1 as response variables, and measures of microbiome alpha (Shannon entropy) and beta (weighted UniFrac) diversity as explanatory variables. Beta diversity was summarized as the first 4 PCs in the PCoA analysis described in Fig. 3A. We also included several other variables that could potentially influence immune measures in the models including age, BMI/BMI categories, gender, location, HIV diagnosis date (indication of how long PLWH were on ART in the ART experienced cohort), and viral load. We used backwards selection [44] to define a set of predictors that associated with immune markers. Models were customized for each cohort as measurements of viral load would not apply to HC and the number of years on ART would not influence the ART-naïve cohort. Models were then applied to each timepoint separately (Fig. 5).

For microbiome explanatory variables, we found that in ART-naïve individuals only, the inflammatory marker IL-6 was negatively associated with PC3 and positively associated with PC4 of the weighted UniFrac PCoA analysis shown in Fig. 3 (Fig. 5). PC1, whose values correlated with having a more Prevotella-rich/Bacteroides-poor microbiome type, positively correlated with CD8 + PD1 + T cells in the ART-naive cohort following 24 weeks of ART treatment (Fig. 5). CD8 + CD38 + HLA-DR + T cells also correlated negatively with PC2 in the HC.

**Fig. 6:**
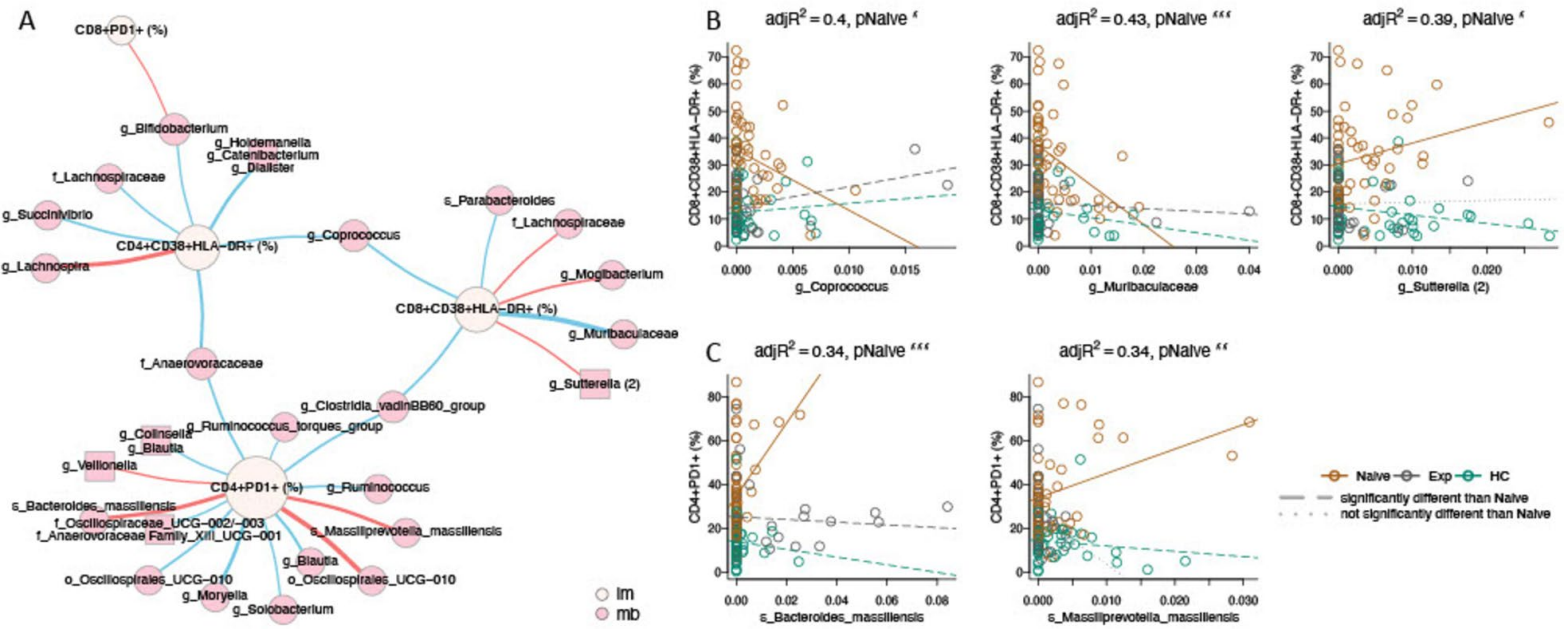
Network analysis of immune and microbial associations. **A** Network summarizing relationships between immune markers (beige nodes) and microbial ASVs (pink nodes) at week 0. Red edges represent positive associations between an immune marker and microbial feature in the ART-naive cohort; blue edges represent negative associations. Edge widths are a function of the *p*-value on the slope of the ART-naive cohort, with thicker edges representing smaller *p*-values. Relationships were generated by linear models of the form immune marker ~ microbial feature + microbial feature × cohort, with an additional term for read count of the microbial feature. Relationships in this network are limited to those with an FDR-adjusted *p*-value on the F statistic of the overall regression < 0.2, adjusted *R*^2^ > 0.25, *p*-value on the slope for the naïve cohort < 0.05 and different from the slope for the experienced cohort and/or the healthy controls (*p* < 0.05), and maximum absolute value of DFFITS < 2. Names are based on Silva taxonomy [43] assignment for each ASV. Square nodes with more than one listed feature represent highly correlated microbes that were binned using SCNIC [46]. **B**, **C** Scatterplots and fitted regression models of associations between CD8 + CD38 + HLA-DR + (**B**), and CD4 + PD1 + (**C**) immune cells and microbial features. Each circle represents one person, colored by cohort. Fitted models for each cohort are shown with colored lines, with dashed lines representing slopes significantly different than naïve (*p* < 0.05), and dotted lines not significantly different than naïve. For all plots, the slope for the naïve cohort is significantly different than zero (pNaive, with significance codes for *p*-values defined as “***” (0, 0.001), “**” (0.001, 0.01), “*” (0.01, 0.05). Adjusted *R*^2^ (adj*R*^2^) is provided as a measure of overall model quality

Other interesting observations for non-microbiome explanatory variables included that BMI negatively correlated with CD4 + CD38 + HLA-DR + T cells in ART-naïve PLWH, and that both age and BMI positively correlated with CRP in HC. Living in the rural versus urban location also influenced immune populations in both the PLWH and HC. CD4 + and CD8 + T cell exhaustion was higher in the rural location compared to urban after 24 weeks of ART (Fig. 5). CD8 + T cell activation (CD38 + HLA-DR +) was also higher in the urban compared to rural area in HC but not HIV-infected cohorts. We next used linear regression to identify individual microbes and modules of highly co-correlated microbes (ASVs defined using DADA2 [45], and modules created with SCNIC [46]) associated with immune factors. We performed separate analyses on data from the baseline and week 24 samples. We then formed a network (Fig. 6), where edges represent relationships between an immune population and microbe where the estimated slope for the HIV-naïve cohort was significantly different from either the ART-experienced or HC cohort. At baseline, this would identify relationships in untreated infection that are corrected with ART or not present in HC. Twenty-nine relationships were identified, with 14 for CD4 + PD1 + cells, 7 for CD8 + CD38 + HLA-DR cells, 7 for CD4 + CD38 + HLA-DR + cells, and 1 for CD8 + PD1 + T cells, and none for the other immune populations tested. Using only baseline data, both negative and positive associations were detected, indicating a potential influence of both protective and detrimental bacteria. When performing the same analysis using only the data from the samples collected at the 24 weeks timepoint, only 2 associations were found, showing a diminishing of these relationships with effective ART.

To evaluate microbe-immune relationships specific to treated infection versus HC, we used a similar approach to identify relationships between ASVs and immune phenotypes that were significantly different than zero for one of the cohorts (either experienced PLWH or HC) and significantly different between the cohorts (see Methods Model M10). These analyses showed a much weaker effect, identifying six relationships at baseline (5 with CD4 + PD1 + cells, and one with CD8 + CD103 + cells) (Figure S12), and 1 relationship at week 24 (specifically, a positive relationship with an ASV in the family *Enterobacteriaceae* (*p*-value of slope in Exp = 4.2 e^−4^).

## Discussion

This study allowed us to assess differences in both immune and gut microbiome response to ART in HIV + individuals living in an urban versus rural area of Zimbabwe and potential relationships between them. Although the percent of PLWH who achieved viral control with ART was not different between the rural and urban locations, improvements in inflammation, T cell activation, and T cell exhaustion markers with successful ART were muted in the rural compared to the urban area. Furthermore, ART with cotrimoxazole prophylaxis had a relatively strong impact on gut microbiome composition compared to HIV infection alone, with reductions in alpha diversity and greater deviation from the microbiome composition of HC. Other important associates with microbiome differences were BMI and living in the rural versus urban location. Finally, we observed significant relationships between gut microbiome composition and inflammation, T cell exhaustion, and chronic immune activation markers particularly in ART-naïve PLWH. Elevated immune exhaustion after 24 weeks of treatment correlated with both living in the rural location and a more Prevotella-rich/Bacteroides-poor microbiome type, suggesting a potential role for microbiome differences or their co-variates in the muted improvements in immune exhaustion in the rural area.

In this study, all participants with HIV-1 were treated with EFV/3TC/TDF. Levels of viral control were consistent with previous reports of an 81% viral control rate with EFV/TDF/FTC [47]; failures have been previously related to both discontinuation due to adverse events and the development of antiviral resistance [47]. That we did not detect a difference in viral control rate between a rural and urban location contrasts with prior studies conducted in SSA that found increased failure of first-line ART in rural locations to be related to poor compliance because of longer travel distances to access health care facilities, increased stigma, and human resources challenges [2, 21, 48]. It is difficult to discern the degree to which the muted improvement in inflammation, T cell activation, and exhaustion observed with successful ART in the rural area could have been related to similar compliance issues.

Non-HIV-related factors that influenced these same immune phenotypes included BMI, gender, and age; however, these factors did not significantly differ in PLWH in the urban and rural locations so are also not likely drivers. The negative correlation between levels of activated CD4 + CD38 + HLA-DR + T cells and BMI in ART-naïve PLWH is consistent with this immune marker and wasting occurring with progression to AIDS [49]. Our data suggests a potential role for microbiome differences that occur with low BMI in CD4 + T cell activation, since BMI significantly affected microbiome composition in our models. It was striking that 43.8% of ART experienced men were in the underweight categories, suggesting that this population may be particularly vulnerable to food insecurity. Our observation of significantly higher CD4 + T cell percent and CD4/CD8 T cell ratio in ART-naïve women compared to men is consistent with the results of several prior studies conducted in SSA, and a sex hormone effect is one possible explanation that has been suggested [50].

Surprisingly, the HC had a significant increase in CD8 + T cells expressing the exhaustion marker PD1 and the ART-experienced cohort in the rural location had an increase in the inflammatory marker CRP over time (Fig. 1) both of which we would have expected to remain unchanged. One potential driver of these changes may be socio-economic change in Zimbabwe during patient sample collection, as indicated by increases in inflation rates, which ranged from 3.52 to 66.8% during baseline sample collections (January 2018 to March 2019) and from 4.29 to 230.54% during 24 week sample collections (July 2018 to August 2019) [37]. In one prior study conducted in Uganda, high levels of inflammatory markers (IL-6 and d-dimer) among PLWH on ART were linked with economic insecurity, specifically a lack of electricity and an unprotected water source [51]. However, we did not see an effect on immune measures of water source (tap, bore hole, or well) in our linear models.

Since one goal of this study was to relate immune markers in HIV to the intestinal microbiome, we also evaluated T cells expressing the mucosal trafficking marker CD103. The chief ligand for CD103 is E-cadherin, a cellular adhesion molecule found on epithelial cells which is important for T cell homing to mucosal sites, including the intestine [52]. Circulating CD103 + T cells share a cellular transcriptome that more closely resembles CD4 + T cells from the gut, suggesting they are homing to the gut [53]. The decrease in CD103 + T cells in ART-naïve PLWH compared to HC could indicate trafficking to the gut to combat HIV-driven challenges. The increase in CD103 + T cells in ART-experienced versus ART-naive could be indicative of a reduction in gut homing of T cells following a partial resolution of mucosal inflammation with ART.

Our study population overall had a relatively Prevotella-rich/Bacteroides-poor microbiome, which is consistent with other studies conducted in SSA [16, 54, 55]. Individuals in the urban area had a significantly different microbiome composition compared to rural, characterized in part by lower values on the PC1 axis that separated Prevotella-rich from Bacteroides-rich microbiomes. This is consistent with prior studies comparing gut microbiomes of individuals in rural versus urban Cameroon and Tanzania [19, 54]. Movement towards a more Bacteroides-rich/Prevotella-poor microbiome has previously been described as a “Westernization” of the microbiome with urbanization [54]. Interestingly, PC1 positively correlated with CD8 + PD-1 + T cell exhaustion in the ART-naïve cohort after 24 weeks of treatment. That elevated immune exhaustion after 24 weeks of treatment correlated with both living in the rural location and a more Prevotella-rich/Bacteroides-poor microbiome type suggests a potential role for microbiome differences or their co-variates in the muted improvements in immune exhaustion in the rural area.

Consistent with prior studies [17], gut microbiome differences with untreated HIV infection were not pronounced, with no significant difference from HC based on beta diversity and only 1 ASV in Clostridium cluster I having a significantly reduced abundance compared to HC. The gut microbiome of ART-naïve PLWH had reduced alpha diversity compared to HC in the rural location, which has been reported previously in SSA [14] and Western countries [27], but inconsistently [17]. Although reduced alpha diversity in untreated HIV infection has previously been linked with disease severity [14], Shannon diversity was not related to CD4 + T cell percent in ART-naïve PLWH in our models. Some ART-naïve PLWH in the rural site were taking the antibiotic cotrimoxazole at baseline; however, time on cotrimoxazole did not correlate with baseline alpha diversity values, suggesting that untreated HIV infection and not cotrimoxazole was driving the reduced alpha diversity observed with untreated HIV infection in the rural area. Microbiome dysbiosis did correlate positively with length on cotrimoxazole in the ART-naïve PLWH at baseline. Cotrimoxazole was previously found to not decrease alpha diversity [56], but to change global composition [57], or suppress potential pathogens such as *Streptococcus* [58], in randomized trials conducted in SSA.

Despite a lack of pronounced microbiome differences in ART naïve PLWH, we did find significant relationships between gut microbiome composition and inflammation; The inflammatory marker IL-6 was negatively associated with PC3 and positively associated with PC4 of the weighted UniFrac PCoA analysis in ART-naïve PLWH only. We also identified many correlations between ASVs and levels of T cell exhaustion and activation that were specific to the ART-naïve PLWH, with CD4 + PD1 + T cells being the strongest hub, followed by CD8 + and CD4 + CD38 + HLA-DR + T cells. ASVs that were positively correlated with these cell populations included bacteria that have been implicated in bacteremia, intra-abdominal or endodontic infections, colorectal cancer, and/or inflammatory bowel diseases including *Bacteroides massiliensis* [59], *Massilliprevotella massiliensis* [60], *Mogibacterium* [61], and *Sutterella* [62, 63]. *B. massiliensis* is additionally a mucin degrader with a preference for host glycans over dietary substrates [64], an activity that has been previously shown to compromise barrier function [65]. *M. massiliensis* has previously been found to correlate with CD8 + CD38 + HLA-DR + T cells in ART-experienced men who have sex with men (MSM) [17]. Many of the negatively correlated bacteria were poorly defined or had unclear effects on health, but included bacteria previously associated with disease protection such as *Parabacteroides* [66], the butyrate producers *Coprococcus* [67], and *Blautia* [68]. *Coprococcus* has been previously associated with improved barrier function among ART-experienced MSM [69]. Taken together, these results suggest that microbiome composition in ART-naïve PLWH may influence levels of immune activation and exhaustion driven by translocation. Although we do not have laboratory validation supporting causality, prior work in our lab showed that the fecal bacteria from ART-naïve PLWH in the USA stimulated high levels CD8 + CD38 + HLA-DR + T cells from peripheral blood mononuclear cells (PBMCs) in vitro and that levels of in vitro stimulation correlated with ex vivo levels of activated CD8 + T cells in matched patient blood [10].

The ART-experienced cohort had more pronounced microbiome differences from the HC. Our results suggest the concomitant use of cotrimoxazole as one driver, since dysbiosis correlated positively with length on cotrimoxazole in the ART-naïve PLWH at baseline. Our observation of reduced alpha diversity in ART-experienced PLWH compared to ART-naïve is consistent with other studies evaluating PLWH exposed to both ART and cotrimoxazole [12] and to PLWH exposed to ART only [70–73], although increased diversity with ART has also been observed [27] and related to longer ART duration [13]. Even within this study, whether alpha diversity was gained or lost with ART depended on baseline values, suggesting differential effects based on the degree of HIV-associated gut microbiome disturbance at the commencement of treatment. Whereas a previous study in Cameroon found a more pronounced effect of ART regimens containing ritonavir-boosted protease inhibitor (PI/r)-based ART compared to NNRTI based regimes [12], one study in Mexico showed reduced diversity with the same ART regimen used here without concomitant cotrimoxazole use, supporting that the loss of diversity observed here may be at least partially driven by the ART drugs themselves [73].

Taxa significantly enriched in ART-experienced PLWH compared to HC include *Megamonas*, *Bilophila*, and *Lachnoclostridium*, which have all been found to be increased in prior studies of gut microbiome differences with treated HIV infection [8, 15, 74]; *Megamonas* has additionally been associated with iron deficiency in the context of treated HIV infection [15], and both *Bilophila* and *Megamonas* can be pro-inflammatory [15, 75]. Taxa that were depleted in ART-experienced PLWH included *Turicibacter*, *Butyrivibrio*, *Blastocystis*, and other poorly defined ASVs in the order Clostridiales. Despite the possibility for microbiome differences observed with ART to be involved in inflammation, we found fewer correlations between the immune readouts and the microbiome within ART-experienced PLWH. Linear models used to identify relationships between individual microbes and immune populations in ART-experienced PLWH identified only a few correlations, again with immune exhaustion. These included positive associations between CD4 + PD1 + T cells and ASVs in genera often considered to be beneficial commensals such as *Bacteroides*, *Faecalibacterium*, and *Gastranaerophilales* (Figure S12).

Our study does have some weaknesses. Since we only used 16S rRNA targeted sequencing of bacteria to assess microbiome composition, our results do not rule out a role for fungi, parasites, viruses, strain level variation, or differences in expressed functions. Furthermore, we were unable to validate immune modulation by fecal bacterial communities using in vitro assays or gnotobiotic mice [34] because we collected fecal samples in a preservative for DNA integrity but not amenable to such functional experiments because of challenges with getting samples from rural Zimbabwe to CO, USA. Due to stigma and concerns for loss of privacy and participant safety [76], we did not collect information on sexual preferences in this cohort. Therefore, we are unable to assess potential confounding resulting from associations between sexual practices and microbiome differences as described in studies of persons with HIV in the USA and Europe [17, 70]. Finally, we were surprised to find differences over time in immune readouts our non-intervention cohorts. Although we suspect that changes in economic stability that occurred over the time of sampling may have been at play, we did not measure factors such as changes in access to food, clean water, or stress over time to confirm drivers. Future analyses of this cohort will also include information on factors including diet and markers of socio-economic status and how they also influence metabolic health.

## Methods

### Recruitment

The rural recruitment site was the Mutoko District Hospital, which is in a small town (population of about 12,500) about a 2-h drive from the city of Harare that services surrounding rural villages. The urban subjects were recruited from the Mabvuku Polyclinic, a large urban clinic administered by the City of Harare. Subjects were excluded from all cohorts if they had used antibiotics (apart from co-trimoxazole) within the prior 2 months, were pregnant, or had a body mass index (BMI) greater than 29 kg/m^2^ (are obese). All participants were 18 years old or older.

### Fecal and blood specimen collection

At the first visit after informed consent was obtained, study participants were given a fecal collection kit. Stool samples were collected in a specimen collector within 24 h prior to their second and third clinic visits, and aliquoted by the study participant into an OmniGene Gut collection system (OM-200, DNA Genotek, Ontario, Canada) for preservation of DNA. A fasting blood sample was collected by venipuncture during the second and third visits. Blood and fecal samples from the rural clinic were couriered to the Infectious Diseases Research Laboratory in the Internal Medicine Unit at the University of Zimbabwe Faculty of Medicine and Health Sciences (Harare), which is a 2-h drive from the Mutoko District Hospital, in a cooler on the day of sample collection and then stored at − 80 °C. Samples for microbiome sequencing were shipped on dry ice to the University of Colorado Anschutz Medical Campus (Aurora, CO) and stored at − 80° C upon arrival.

### Collection of demographic information

Study participants filled out a questionnaire which included questions on age, body mass index (BMI), gender, education level, water source, and factors related to occupation and home life.

### Blood sample processing

A subset of the blood sample was analyzed by flow cytometry using fresh PBMCs at the Infectious Diseases Research Laboratory (IDRL) located in the Internal Medicine Unit at the University of Zimbabwe Faculty of Medicine and Health Sciences. Whole blood was collected in BD vacutainer tubes containing sodium heparin and red blood cells (RBCs) from 500 mL of blood were lysed with 1 × RBC lysis Buffer (Thermo Fisher). Cells were washed twice with staining buffer containing PBS, 2% BSA, 1 mM EDTA, and surface stained with BV785-labeled anti-CD3 antibody (BioLegend Cat# 317330), PerCP/Cy5.5-labeled anti-CD4 (BioLegend Cat# 317428), BV421-labeled anti-CD8 antibody (BioLegend Cat# 344748), BV605 labeled anti-CD38 antibody (BioLegend Cat# 303533), Pe-labeled anti-CD103 antibody (BioLegend Cat# 350206), Pe-Cy7 labeled anti-HLA-DR antibody (BioLegend Cat# 307616), and FITC-labeled anti-PD-1 antibody (BioLegend Cat# 329904) or appropriate fluorescence minus one (FMO) controls. Cells were washed twice with staining buffer and fixed in 1% formaldehyde. Cells were acquired on a BD LSRFortessa flow cytometer and analyzed by FlowJo. Representative staining and gating for CD103, PD1, and T cell activation (CD38 + HLA-DR +) on CD4 + and CD8 + T cells is shown in Figure S13.

Plasma was isolated and frozen for shipment to the University of Colorado for measurement of CRP and IL-6 with ELISA following the manufacturer’s protocol (CRP: R&D Systems cat. DCRP00, IL-6 Invitrogen cat. 88–7066).

Blood samples were also used to evaluate absolute CD4 + T cell count or CD4 + T cell percent using the Sysmex (formally Partec) CyFLOW cytometer, and CD4 easy count kit or CD4 percent easy count kit following manufacturer’s instructions (Sysmex, cat: 058401, 058505, respectively), and HIV viral load using a Roche COBAS AmpliPrep/COBAS TaqMan (CAP/CTM) instrument with COBAS AmpliPrep/TaqMan HIV-1 test v2.0 kit following manufacturer’s instructions at the Infectious Diseases Research Laboratory (IDRL) located in the Internal Medicine Unit, UZ Faculty of Medicine and Health Sciences (UZFMHS).

### DNA extraction and sequencing

DNA was extracted using the DNeasy PowerSoil Kit protocol (Qiagen). Extracted DNA was PCR-amplified with barcoded primers targeting the V4 region of 16S rRNA gene according to the Earth Microbiome Project 16S Illumina Amplicon protocol with the 515F:806R primer constructs [77]. A sterile water blank was included in each batch of extractions and PCR amplification to serve as a procedural control. Each PCR product was quantified using PicoGreen (Invitrogen), and equal amounts (ng) of DNA from each sample were pooled and cleaned using the UltraClean PCR Clean-Up Kit (MoBio). Sequences were generated on two runs on a MiSeq personal sequencer (Illumina, San Diego, CA).

### Software

Unless otherwise specified, all analyses were run in R version 4.2.2 (2022–10-31) [78]. 

### Data preprocessing

Before analyses were performed, one sample with no immune data (ZIM033.3) was removed. ART-naïve patients at week 24 and ART experienced patients at week 0 were labeled as “viremic” if they had more than 200 copies of HIV/mL of blood [23]. There were 10 patients whose CD4 T cell immune data was not able to be measured. Therefore, we imputed values for that missing data, specifically, for the following immune markers: CD4 + CD38 + HLA-DR + (%), CD4 + PD1 + (%), CD4 + CD103 + (%). Imputed values were calculated as the average immune marker value. Length on ART was calculated for the ART-experienced PLWH as the number of days between the visit date and the date that they started ART. For all longitudinal analyses, only study participants who provided samples at both baseline and week 24 were included.

### Immune phenotype analyses

#### Fixed-effects ordinary least squares linear modeling

Linear modeling performed in Figure S6 (M1) used the *feols* function in the *fixest* package [79] in R to run fixed-effects OLS linear modeling.

M1 $$Immune\;marker\sim Cohort+Week+Cohort\ast Week$$

Model (M1) was run on all study participants and then stratified by location and clustered by Person ID (PID), due to the longitudinal aspect of the study. *ggplot* [80] was then used to visualize results. Immune marker values were not imputed for this analysis.

#### Inter- and intra-cohort immune analyses

For the inter-cohort tests (Fig. 1), all baseline values were used regardless of whether the study participant completed the 24 week visit. ART-experienced individuals who were viremic at baseline were excluded to evaluate the effects of successful ART. Significant differences were assessed with a Kruskal–Wallis test with a Dunn’s post hoc. In the intra-cohort longitudinal comparisons, study participants who did not complete both the baseline and 24 week visit were excluded as were all viremic patients (including ART-naïve and experienced). Significant differences were assessed with a paired Mann–Whitney *U* test. No immune marker means were imputed in this analysis. *ggplot* [80] was used to visualize results.

### Microbiome analyses

#### Core metrics analysis and taxonomic classification

Demultiplexing of 16S rDNA gene sequences and quality control using DADA2 [45] to define ASVs were performed in QIIME2 (version 2023.5) [81]. SILVA (version 138) [82, 83] was used to perform taxonomic classification of each ASV. Taxa that were not classified at the phylum level or that were classified as mitochondria and chloroplasts were excluded. SEPP [84] was used to produce a phylogeny of ASVs for use in diversity analyses. The feature table of ASVs was rarified to a sampling depth of 16,645 sequences per sample prior to downstream analyses.

Alpha diversity, measured as Shannon entropy [38], was plotted across all three cohort and over time (Fig. 2A, B) using QIIME 2. Linear modeling (Fig. 2C) (M2) was performed using the *lm* function in stats package [78].

M2 $$\Delta Shannon\;Entropy\sim Baseline\;Shannon\;Entropy$$

Beta diversity, measured using weighted UniFrac distances [40], was calculated using the core metrics functions in QIIME2. Predictors were found to be correlated with weighted UniFrac PCoA arms using mixed effects linear modeling (Table S3).

M3 $$PCoA\;Arm\sim All\;Predictors+(1\vert PID)$$

Weighted UniFrac PCoA results from the core metrics analysis were used to calculate coordinates for biplots in QIIME2 and then visualized in *R*. To evaluate potential moderators of beta diversity, two different Adonis tests [85] were performed in R. The models that were run were:

M4 $$Weighted\;UniFrac\sim Age+HIV\;Status+Treatment\;Status$$

M5 $$Weighted\;UniFrac\sim Location+Week+Location\ast Week$$

Both models M4 and M5 were stratified (using the strata parameter) by PID to account for the dependence between samples from the same patient. Age was included in model M4 as a potential confounder since it significantly differed across cohorts. Dysbiosis was calculated in R as the mean distance of each sample in the ART-naïve and experienced cohort to all healthy controls of the same timepoint. Linear modeling (M6) was then performed using the *lm* function in stats package [78] for each cohort and each location separately (Fig. 3B, C).

M6 $$\Delta Dysbiosis\sim Baseline\;Dysbiosis$$

#### Compositional differences

To determine genera that significantly differed in abundance across cohorts, we used ANCOM-BC (version 2.0.2) [41]. This allowed us to account for the compositional nature of microbiome data, control for location and viral control with ART as potential confounders, and account for the longitudinal study design. Analysis was performed in R using the Phyloseq (version 1.42.0) [86], and Qiime2R (version 0.99.6) [87] packages. The taxonomic level that we evaluated at was genus and the reference group used in the analysis was the healthy controls. Subsequently, three models were tested using ANCOM-BC.

M7 $$Filtered\;Taxa\sim Location+Viral\;Outcome+Cohort$$

Model M6 was run only on baseline values. Also, values were grouped by cohort which is used to help detect structural zeros and performing multi-group comparisons. Results are shown in Fig. 4A.

M8 $$Filtered\;Taxa\sim Location+Week+Viral\;Outcome+Cohort+\left(1\right|PID)$$

Model M7 evaluated values at both timepoints (both week 0 and week 24) and grouped by cohort as in model M6. The random effects term was added to account for the dependence between samples of the same patient. Results are shown in Fig. 4B, and it should be noted that since the second timepoint was after 24 weeks of ART/cotrimoxazole, these additional differences from baseline also will reflect changes with ART.

M9 $$Filtered\;Taxa\sim Location+Week+Viral\;Outcome+(1\vert PID)$$

Model M8 evaluated values at both timepoints but only for those patients who were ART-naïve PLWH at baseline, to evaluate changes over time with ART. No results were visualized for this model as there were very few differences. For models M6 and M7, visualizations (Fig. 4A, B) were produced using *ggplot* [80] and *microViz* [88].

### Integrative analysis of immune markers and microbiome

#### Predictive models for immune markers

To understand the impact that other variables might have on patient immunity (Fig. 5), we curated a list of potential clinical and demographic features: cohort, location, gender, BMI, BMI categories (severe thinness, moderate thinness, mild thinness, normal, overweight, and obese), HIV status (negative/positive), week, age, Bristol stool score, education level (primary, secondar, and tertiary), water source (tap, bore hole, and well), cotrimoxazole (Y/N), length on cotrimoxazole (months), normal work transportation (bus or train, car or motorcycle, does not travel to work, and walk or bike), manual work, manual chores at home (Y/N), head of household (Y/N), and length on ART (years); and measures of microbial community diversity (PCA1, PCA2, PCA3, PCA4 from the weighted UniFrac PCoA and Shannon entropy). Since linear models inherently penalize large numbers of explanatory variables [89] because more variables in the model can reduce results accuracy due to overfitting, we reduced the number of explanatory variables when developing the models using backwards stepwise regression feature selection [44]. Overall, 226 samples were evaluated—including individuals who had come in at both timepoints and who had controlled infection—against the 24 aforementioned explanatory variables for each immune marker. Afterwards, viral load was included in the models pertaining to PLWH (naïve and experienced); and HIV diagnosis date was included in models pertaining only to ART-experienced PLWH. Models were created for each immune marker, cohort, and time separately using the *lm* function from stats package [78].

#### Network analysis of immune and microbial associations

Data for 8 immune markers and 237 microbial features were combined in a dataset spanning 127 participants at baseline and 113 participants at week 24. Analyses were run separately for baseline and week 24 data. Microbial features were limited to those observed in > 20% of samples, resulting in 191 single ASVs and 46 modules (Table S4) of highly co-correlated ASVs aggregated by SCNIC using default parameters [46]. Features were expressed as relative abundance. For each timepoint, linear regressions were performed for each pair of immune markers and microbial features as previously described [90]. The form of the model was:

M10 $$Immune\;Marker\sim Microbial\;Read\;Count+Microbial\;Feature+Microbial\;Feature\ast Cohort$$

The read count term accounted for differences in across samples since this analysis did not use rarefied data. To identify relationships that were evident in ART-naïve PLWH and different from ART-experienced PLWH or HC, resulting models were filtered to include only those with an FDR-adjusted *p*-value on the F statistic of the overall regression < 0.2, adjusted *R*^2^ > 0.25, *p*-value on the slope for the ART-naïve cohort < 0.05 and different from the slope for the ART-experienced cohort and/or the HC (*p* < 0.05 for at least one of these slopes), and maximum absolute value of DFFITS < 2 (to exclude results that were outlier driven). The resulting network and models were visualized using the VOLARE [90] web application. A high-resolution version of the network was generated with the *igraph* [91] library in *R*. To compare microbe:immune relationships observed in ART-experienced PLWH to HC, data was filtered to exclude the ART naïve cohort. Resulting models were limited to those with FDR-adjusted *p*-value on the F statistic of the overall regression < 0.2, adjusted *R*^2^ > 0.25, one of the slopes for the 2 cohorts different than 0, and the slopes for the 2 cohorts being different than each other ((pSlopeExp < 0.05 | pSlopeHC < 0.05) and pExp_v_HC < 0.05), maximum absolute value of DFFITS < 2.

### Supplementary Information

**Additional file 1: Figure S1.** Number of study participants and samples used in different analyses and excluded for different reasons. **Figure S2.** CD4+ T cell percent and CD4/CD8 percent ratios across all cohorts stratified by location and across time (Baseline= Week 0). Statistical significance was calculated using a paired Mann-Whitney U test. *P*-values are coded as ‘****’ between [0, 0.0001], ‘***’ (0.0001, 0.001], ‘**’ (0.001, 0.01], ‘*’ (0.01, 0.05], with square brackets indicating that the endpoints are included in the interval. **Figure S3.** Detailed plots of CD8+CD38+HLA-DR+ (Panels A, B) and CD4+CD38+HLA-DR+ T (Panels C, D) cells underlying *p*-values reported in Fig. 1A. Panels A, C: Inter-cohort comparisons using only baseline (Week 0) values. Statistical significance assessed using a Kruskal Wallis with a Dunn’s post-hoc test. Panels B,D: Intra-cohort longitudinal comparisons over time. Statistical significance assessed using a paired Mann-Whitney U test. *P*-values are coded as ‘****’ between [0, 0.0001], ‘***’ (0.0001, 0.001], ‘**’ (0.001, 0.01], ‘*’ (0.01, 0.05], with square brackets indicating that the endpoints are included in the interval. **Figure S4.** Detailed plots of CD8+PD1+ T cells (Panels A, B) and CD4+PD1+ T cells (Panels C, D) underlying *p*-values reported in Fig. 1A. Panels A, C: Inter-cohort comparisons using only baseline (Week 0) values. Statistical significance assessed using a Kruskal Wallis test with a Dunn’s post-hoc test. Panels B,D: Intra-cohort longitudinal comparisons over time. Statistical significance assessed using a paired Mann-Whitney U test. *p*-values are coded as ‘****’ between [0, 0.0001], ‘***’ (0.0001, 0.001], ‘**’ (0.001, 0.01], ‘*’ (0.01, 0.05], with square brackets indicating that the endpoints are included in the interval. **Figure S5.** Detailed plots of IL-6 (Panels A, B) and CRP (Panels B, C) levels underlying *p*-values reported in Fig. 1A. Panels A,C: Inter-cohort comparisons using only baseline (Week 0) values. Statistical significance assessed using a Kruskal Wallis with a Dunn’s post-hoc test. Panels B,D: Intra-cohort longitudinal comparisons over time. Statistical significance assessed using a paired *P*-values are coded as ‘****’ between [0, 0.0001], ‘***’ (0.0001, 0.001], ‘**’ (0.001, 0.01], ‘*’ (0.01, 0.05], with square brackets indicating that the endpoints are included in the interval. *p*-values < 0.0001 are labeled as ****, < 0.001 are ***, 0.01 are **, and < 0.05 are *. **Figure S6.** Detailed plots of CD8+CD103+ (Panels A, B) and CD8+CD103+ (Panels C, D) T cells underlying *p*-values reported in Fig. 1A. Panels A, C: Inter-cohort comparisons using only baseline (Week 0) values. Statistical significance assessed using a Kruskal Wallis with a Dunn’s post-hoc test. Panels B, D: Intra-cohort longitudinal comparisons over time. Statistical significance assessed using a paired Mann-Whitney U test. *P*-values are coded as ‘****’ between [0, 0.0001], ‘***’ (0.0001, 0.001], ‘**’ (0.001, 0.01], ‘*’ (0.01, 0.05], with square brackets indicating that the endpoints are included in the interval. **Figure S7.** Fixed-effects ordinary least squares (OLS) linear modeling was used to evaluate the combined effects of differences in cohorts and time points overall (left panel) and stratified by rural (middle panel) and urban (right panel) location. From the left, columns representing predictors should be interpreted as follows: the effect of ART naïve PLWH relative to healthy controls; the effect of ART experienced PLWH relative to heathy controls; change over time (COT) in healthy controls; the interaction of ART naïve PLWH with time relative to healthy controls and time; and the interaction of ART experienced PLWH with time relative to healthy controls and time. Coefficient and color show directionality of the relationship – red is a positive effect and blue negative. *P*-values are coded as ‘****’ between [0, 0.0001], ‘***’ (0.0001, 0.001], ‘**’ (0.001, 0.01], ‘*’ (0.01, 0.05], with square brackets indicating that the endpoints are included in the interval. **Figure S8.** Correlation between the Length of time on Cotrimoxazole (in months) at Baseline (week 0) and alpha diversity (Shannon Entropy) and Dysbiosis (average weighted UniFrac distance of a given sample from each of the health control samples). Values shown for only the ART naïve cohort. A linear regression model was used with an interaction term for rural versus urban location. **Figure S9.** Taxa bar plots. Taxonomic assignments were made using a QIIME2 trained naïve-bayes classifier and the Silva (version 138) taxonomic database [51].  Each color represents a different bacterial genus, and genera within the same Phylum are depicted in different shades of the same color using microshades [92]. Samples are stratified by cohort and time. **Figure S10.** Weighted UniFrac Principal Coordinates Analysis (PCoA). The PCoA was conducted using data from all of the samples, but only a subset of samples are plotted in the different quadrants depending on whether they were from the rural or urban location (columns) or collected at week 0 or week 24 (rows). Points are colored by cohort. Naïve = HIV+ ART Naïve cohort, Exp = HIV+ ART Experienced cohort, HC= Healthy controls. The percent of variation explained by PC1 and PC2 are indicated on the x and y axes respectively. **Figure S11.** Detailed plots of microbiome dysbiosis levels. A dysbiosis value for each sample was calculated as the average weighted UniFrac distance of that sample from each of the health control samples. Panel A: Inter-cohort comparisons using only baseline (Week 0) values. Statistical significance assessed using a Mann Whitney U test. Panel B: Intra-cohort longitudinal comparisons over time. Statistical significance was assessed using a paired Mann-Whitney U test. *P*-values are coded as ‘****’ between [0, 0.0001], ‘***’ (0.0001, 0.001], ‘**’ (0.001, 0.01], ‘*’ (0.01, 0.05], with square brackets indicating that the endpoints are included in the interval. **Figure S12.** (A) Network summarizing relationships between immune markers (beige nodes) and microbial ASVs (dark pink nodes) at Week 0 for only 2 cohorts: ART experienced and healthy controls. Red edges represent positive associations between an immune marker and microbial feature in the ART Naive cohort. Edge widths are a function of the *p*-value on the slope of the ART Naive cohort, with thicker edges representing smaller *p*-values. Relationships were generated by linear models of the form immune marker ~ microbial feature + microbial feature x cohort, with an additional term for read count of the microbial feature. Relationships in this network are limited to those with an FDR-adjusted *p*-value on the F statistic of the overall regression < 0.2, adjusted R2 > 0.25, *p*-value on the slope for the Naïve cohort < 0.05 and different from the slope for the Experienced cohort and/or the healthy controls (*p*<0.05), and maximum absolute value of DFFITS < 2. Names are based on Silva taxonomy assignment for each ASV. Square nodes with more than one listed feature represent highly correlated microbes that were binned using SCNIC. B, C) Scatterplots and fitted regression models of associations between CD4+PD1+ (B), and CD8+PCD103+ (C) immune cells and microbial features.  Each circle represents one person, colored by cohort (brown=experienced, green=healthy control).  Fitted models for each cohort are shown with colored lines, with dashed lines representing slopes significantly different than 0 (*p* < 0.05), and dotted lines not significantly different than 0. For all plots, the slope for the experienced cohort is significantly different than zero (pExp, with significance codes for *p*-values defined as ‘***’ [0, 0.001], ‘**’ (0.001, 0.01], ‘*’  (0.01, 0.05); where square brackets indicate endpoints included in the interval).  Adjusted R2 (adjR2) is provided as a measure of overall model quality. **Figure S13.** Representative staining and gating for CD103, PD1 and T cell activation (CD38+HLA-DR+) on CD4+ and CD8+ T cells in human PBMC. Human PBMC was stained for CD3, CD4, CD8, CD38, CD103, HLA-DR and PD-1 and analyzed by flow cytometry. CD3+ T cells were gated through singlet and lymphocyte gates. CD4+ and CD8+ T cells were then examined for expression of CD103, PD-1 and activation as determined by double positive expression of CD38 and HLA-DR (top right quadrant). Staining of PBMC from a HIV seronegative participant is shown. **Table S1.** Clinical and demographic characteristics of study population by cohort at baseline visit, stratified by sex. *P*-values were calculated using Mann Whitney U test. NA represents values not collected/ relevant to a particular cohort. Values are reported as the median with the minimum and maximum range indicated in brackets. Virologic failure is defined as PLWH who are on ART but have uncontrolled viral replication(> 200 copies of HIV per milliliter of blood) [23, 24]. BMI categories were determined using World Health Organization (WHO) standards [25]. **Table S2.** Clinical and demographic characteristics of study population by cohort at baseline visit, stratified by the rural versus urban location. *P*-values were calculated using Mann Whitney U test. NA represents values not collected/relevant to a particular cohort. Values are reported as the median with the minimum and maximum range indicated in brackets. BMI categories were determined using World Health Organization (WHO) standards [25]. **Table S3.** Mixed effect linear model *p*-value results with Weighted UniFrac PCoA arms as response variables. Location decreases from Rural to Urban and ART status increases from no treatment to on treatment. For continuous variables blue indicates negative correlation and red positive correlation. **Table S4.** Composition of largest modules found using SCNIC. Modules will have over four ASVs assigned to them.

## Data Availability

The 16S rRNA data is available at EBI (project PRJEB66206, accession ERP151280). An R markdown file and corresponding data can also be found on Zenodo at https://zenodo.org/record/8346401. To visualize the networks shown in Fig. 6 and Figure S12 and the underlying scatter plots supporting each edge, we have also made available the files *Volare_Zimbabwe_W0_NaiveExpHC.json and Volare_Zimbabwe_W0_ExpHC.json* in the Zenodo repository. These files can be uploaded to a web hosted version of VOLARE found at http://aasix.cytoanalytics.com/volare/.
