# Supplementary material for: Differential effects of antiretroviral treatment on immunity and gut microbiome composition in people living with HIV in rural versus urban Zimbabwe
Source: Microbiome. 2024 Feb 3;12:18. doi: 10.1186/s40168-023-01718-4 (PMC10837999; doi:10.1186/s40168-023-01718-4)
Supplement: Supplementary file 2 — Additional file 1: Figure S1. Number of study participants and samples used in different analyses and excluded for different reasons. Figure S2. CD4+ T cell percent and CD4/CD8 percent ratios across all cohorts stratified by location and across time (Baseline= Week 0). Statistical significance was calculated using a paired Mann-Whitney U test. P-values are coded as ‘****’ between [0, 0.0001], ‘***’ (0.0001, 0.001], ‘**’ (0.001, 0.01], ‘*’ (0.01, 0.05], with square brackets indicating that the endpoints are included in the interval. Figure S3. Detailed plots of CD8+CD38+HLA-DR+ (Panels A, B) and CD4+CD38+HLA-DR+ T (Panels C, D) cells underlying p-values reported in Fig. 1A. Panels A, C: Inter-cohort comparisons using only baseline (Week 0) values. Statistical significance assessed using a Kruskal Wallis with a Dunn’s post-hoc test. Panels B,D: Intra-cohort longitudinal comparisons over time. Statistical significance assessed using a paired Mann-Whitney U test. P-values are coded as ‘****’ between [0, 0.0001], ‘***’ (0.0001, 0.001], ‘**’ (0.001, 0.01], ‘*’ (0.01, 0.05], with square brackets indicating that the endpoints are included in the interval. Figure S4. Detailed plots of CD8+PD1+ T cells (Panels A, B) and CD4+PD1+ T cells (Panels C, D) underlying p-values reported in Fig. 1A. Panels A, C: Inter-cohort comparisons using only baseline (Week 0) values. Statistical significance assessed using a Kruskal Wallis test with a Dunn’s post-hoc test. Panels B,D: Intra-cohort longitudinal comparisons over time. Statistical significance assessed using a paired Mann-Whitney U test. p-values are coded as ‘****’ between [0, 0.0001], ‘***’ (0.0001, 0.001], ‘**’ (0.001, 0.01], ‘*’ (0.01, 0.05], with square brackets indicating that the endpoints are included in the interval. Figure S5. Detailed plots of IL-6 (Panels A, B) and CRP (Panels B, C) levels underlying p-values reported in Fig. 1A. Panels A,C: Inter-cohort comparisons using only baseline (Week 0) values. Statistical signi [file 40168_2023_1718_MOESM1_ESM.docx]

# Supplemental Figures


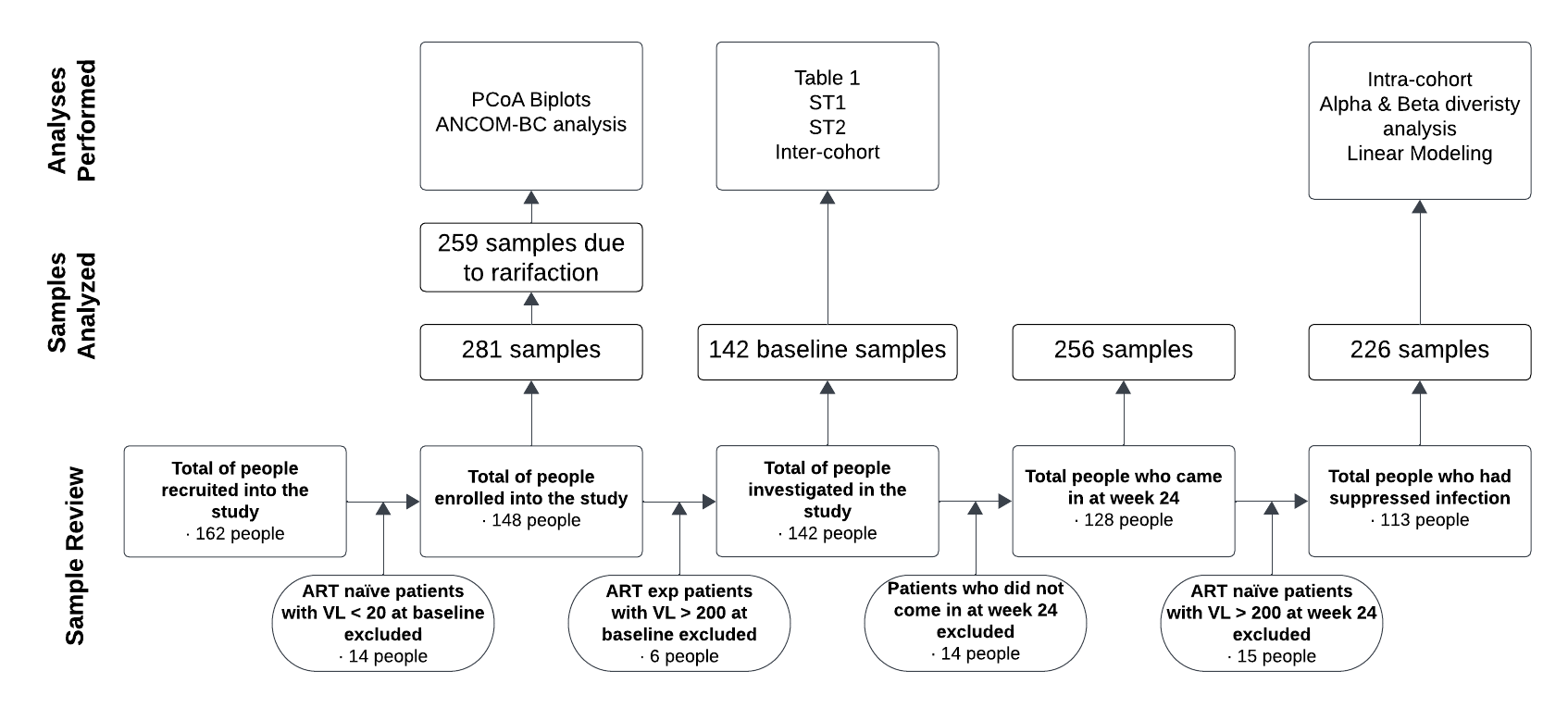


Figure S1: Number of study participants and samples used in different analyses and excluded for different reasons.


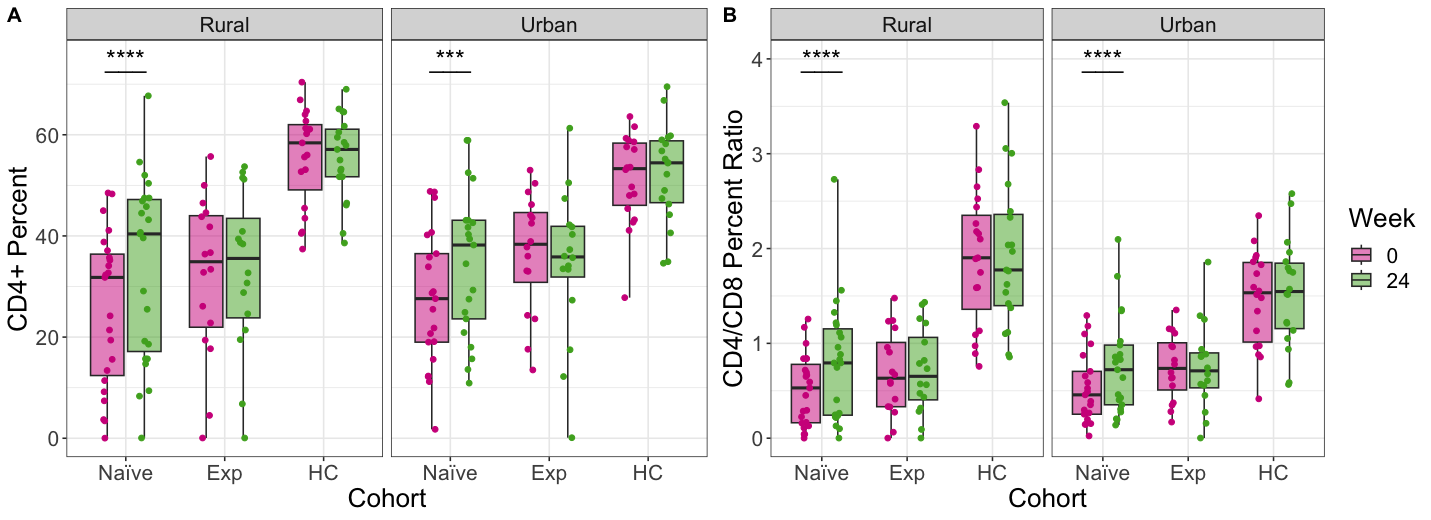


Figure S2: CD4+ T cell percent and CD4/CD8 percent ratios across all cohorts stratified by location and across time (Baseline= Week 0). Statistical significance was calculated using a paired Mann-Whitney U test. P-values are coded as ‘****’ between [0, 0.0001], ‘***’ (0.0001, 0.001], ‘**’ (0.001, 0.01], ‘*’ (0.01, 0.05], with square brackets indicating that the endpoints are included in the interval.


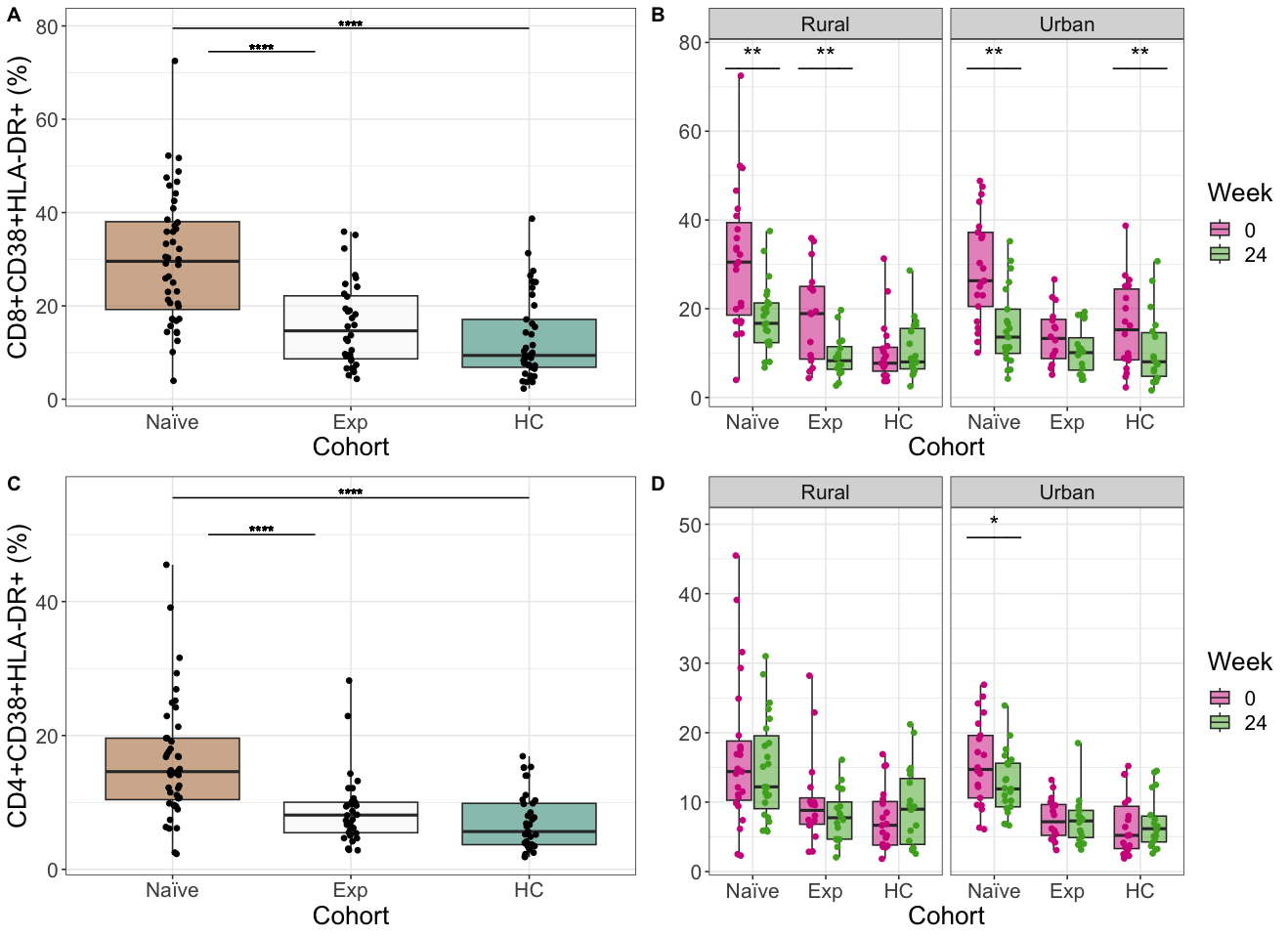


Figure S3: Detailed plots of CD8+CD38+HLA-DR+ (Panels A,B) and CD4+CD38+HLA-DR+ T (Panels C,D) cells underlying p-values reported in Figure 1A. Panels A,C: Inter-cohort comparisons using only baseline (Week 0) values. Statistical significance assessed using a Kruskal Wallis with a Dunn’s post-hoc test. Panels B,D: Intra-cohort longitudinal comparisons over time. Statistical significance assessed using a paired Mann-Whitney U test. P-values are coded as ‘****’ between [0, 0.0001], ‘***’ (0.0001, 0.001], ‘**’ (0.001, 0.01], ‘*’ (0.01, 0.05], with square brackets indicating that the endpoints are included in the interval.


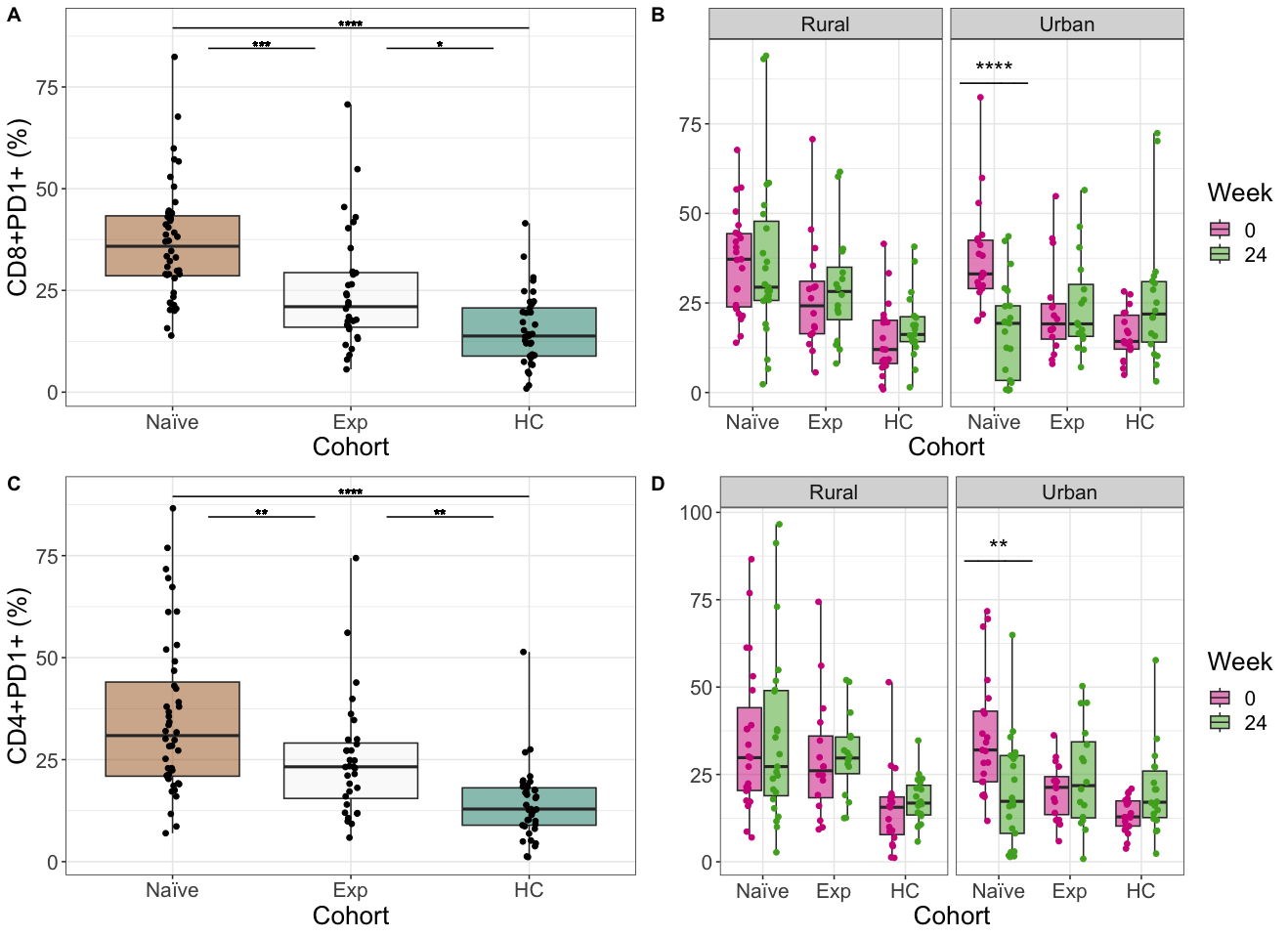


Figure S4: Detailed plots of CD8+PD1+ T cells (Panels A,B) and CD4+PD1+ T cells (Panels C, D) underlying p-values reported in Figure 1A. Panels A,C: Inter-cohort comparisons using only baseline (Week 0) values. Statistical significance assessed using a Kruskal Wallis test with a Dunn’s post-hoc test. Panels B,D: Intra-cohort longitudinal comparisons over time. Statistical significance assessed using a paired Mann-Whitney U test. P-values are coded as ‘****’ between [0, 0.0001], ‘***’ (0.0001, 0.001], ‘**’ (0.001, 0.01], ‘*’ (0.01, 0.05], with square brackets indicating that the endpoints are included in the interval.


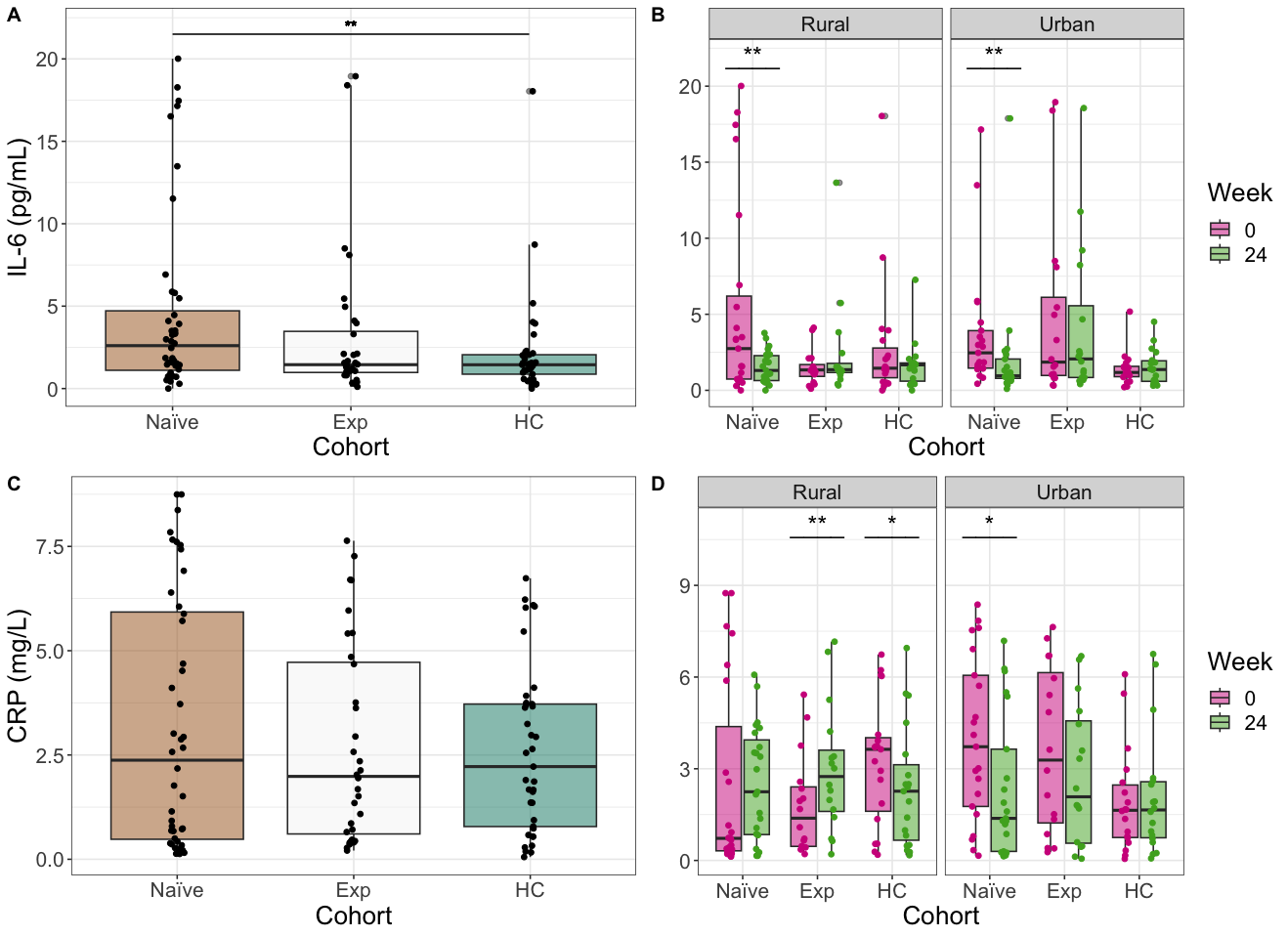


Figure S5: Detailed plots of IL-6 (Panels A,B) and CRP (Panels B,C) levels underlying p-values reported in Figure 1A. Panels A,C: Inter-cohort comparisons using only baseline (Week 0) values. Statistical significance assessed using a Kruskal Wallis with a Dunn’s post-hoc test. Panels B,D: Intra-cohort longitudinal comparisons over time. Statistical significance assessed using a paired P-values are coded as ‘****’ between [0, 0.0001], ‘***’ (0.0001, 0.001], ‘**’ (0.001, 0.01], ‘*’ (0.01, 0.05], with square brackets indicating that the endpoints are included in the interval.p-values < 0.0001 are labeled as ****, < 0.001 are ***, 0.01 are **, and < 0.05 are *.


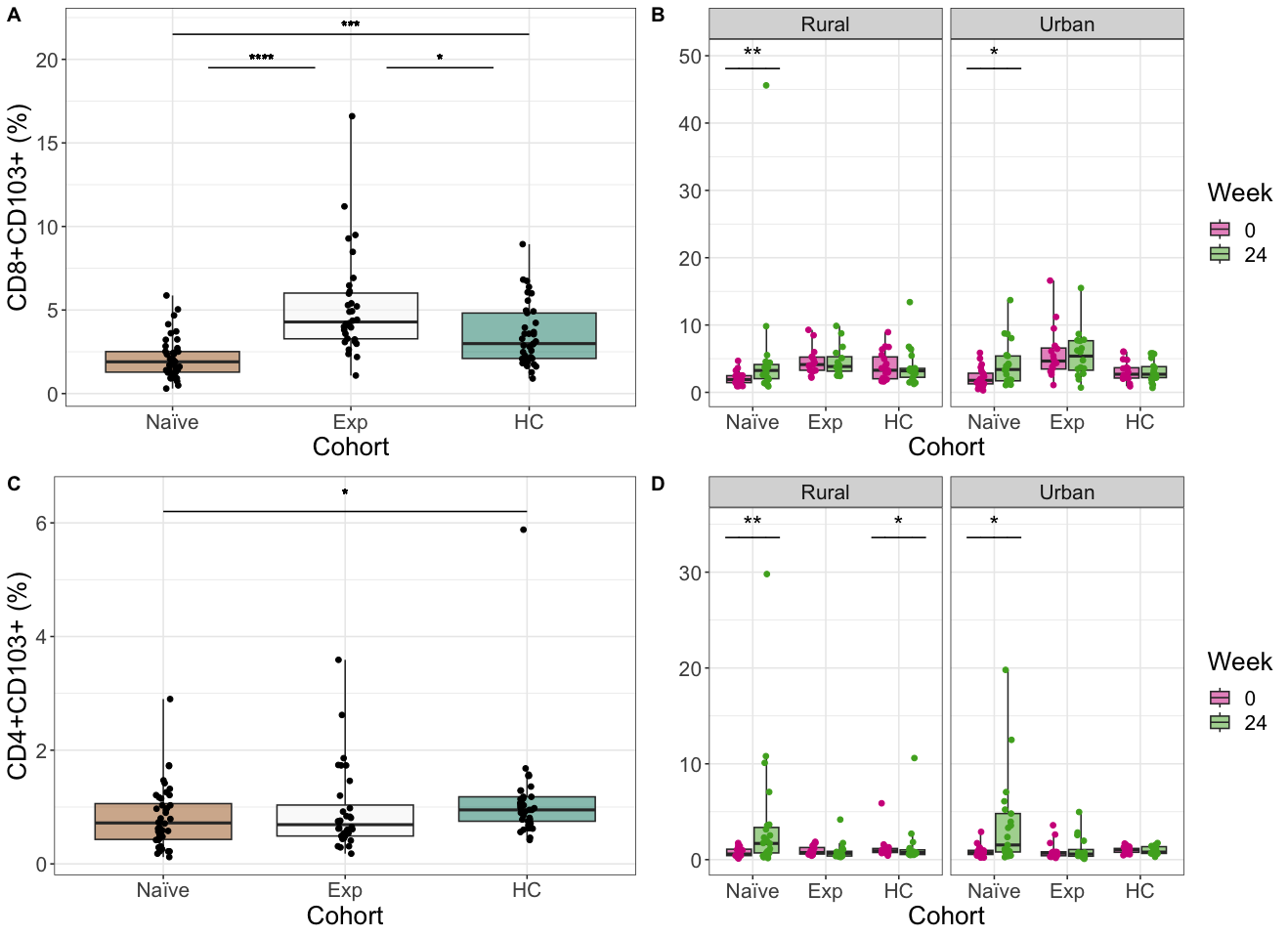


Figure S6: Detailed plots of CD8+CD103+ (Panels A,B) and CD8+CD103+ (Panels C,D) T cells underlying p-values reported in Figure 1A. Panels A,C: Inter-cohort comparisons using only baseline (Week 0) values. Statistical significance assessed using a Kruskal Wallis with a Dunn’s post-hoc test. Panels B,D: Intra-cohort longitudinal comparisons over time. Statistical significance assessed using a paired Mann-Whitney U test. P-values are coded as ‘****’ between [0, 0.0001], ‘***’ (0.0001, 0.001], ‘**’ (0.001, 0.01], ‘*’ (0.01, 0.05], with square brackets indicating that the endpoints are included in the interval.


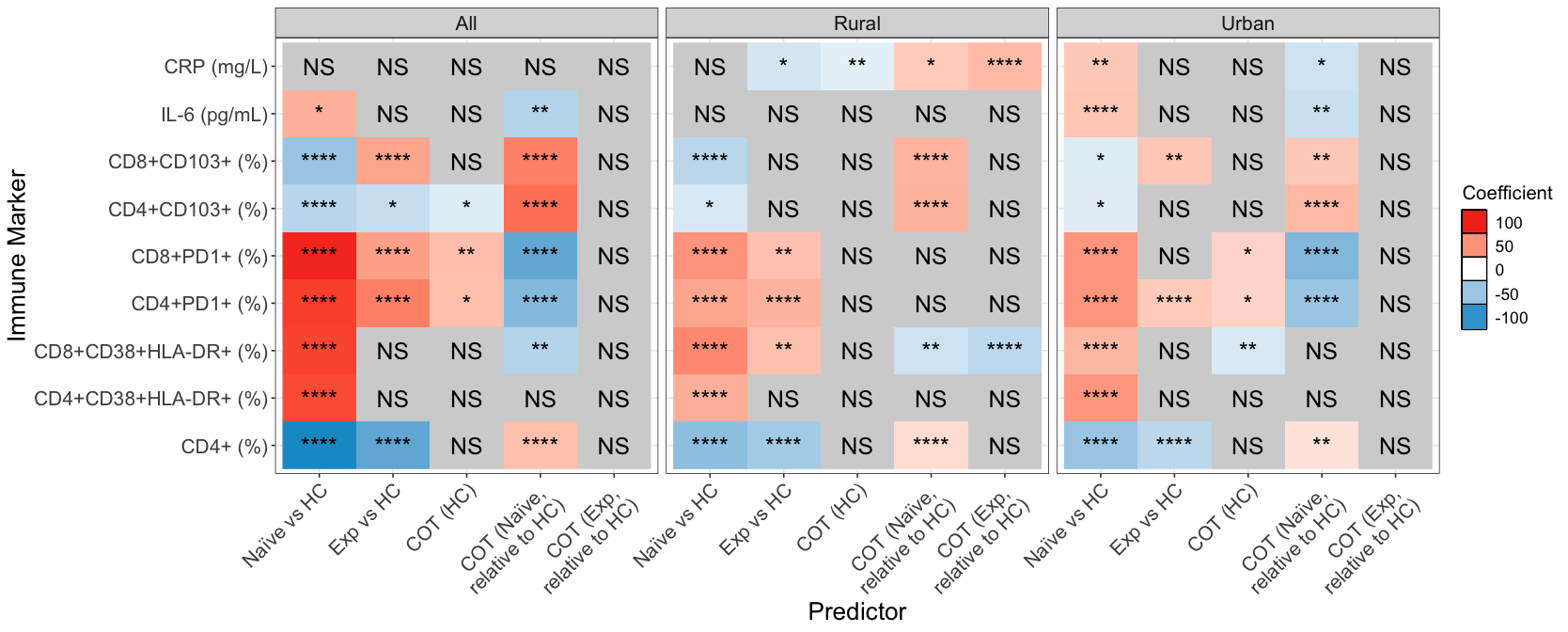


Figure S7: Fixed-effects ordinary least squares (OLS) linear modeling was used to evaluate the combined effects of differences in cohorts and time points overall (left panel) and stratified by rural (middle panel) and urban (right panel) location. From the left, columns representing predictors should be interpreted as follows: the effect of ART naïve PLWH relative to healthy controls; the effect of ART experienced PLWH relative to heathy controls; change over time (COT) in healthy controls; the interaction of ART naïve PLWH with time relative to healthy controls and time; and the interaction of ART experienced PLWH with time relative to healthy controls and time. Coefficient and color show directionality of the relationship – red is a positive effect and blue negative. P-values are coded as ‘****’ between [0, 0.0001], ‘***’ (0.0001, 0.001], ‘**’ (0.001, 0.01], ‘*’ (0.01, 0.05], with square brackets indicating that the endpoints are included in the interval.


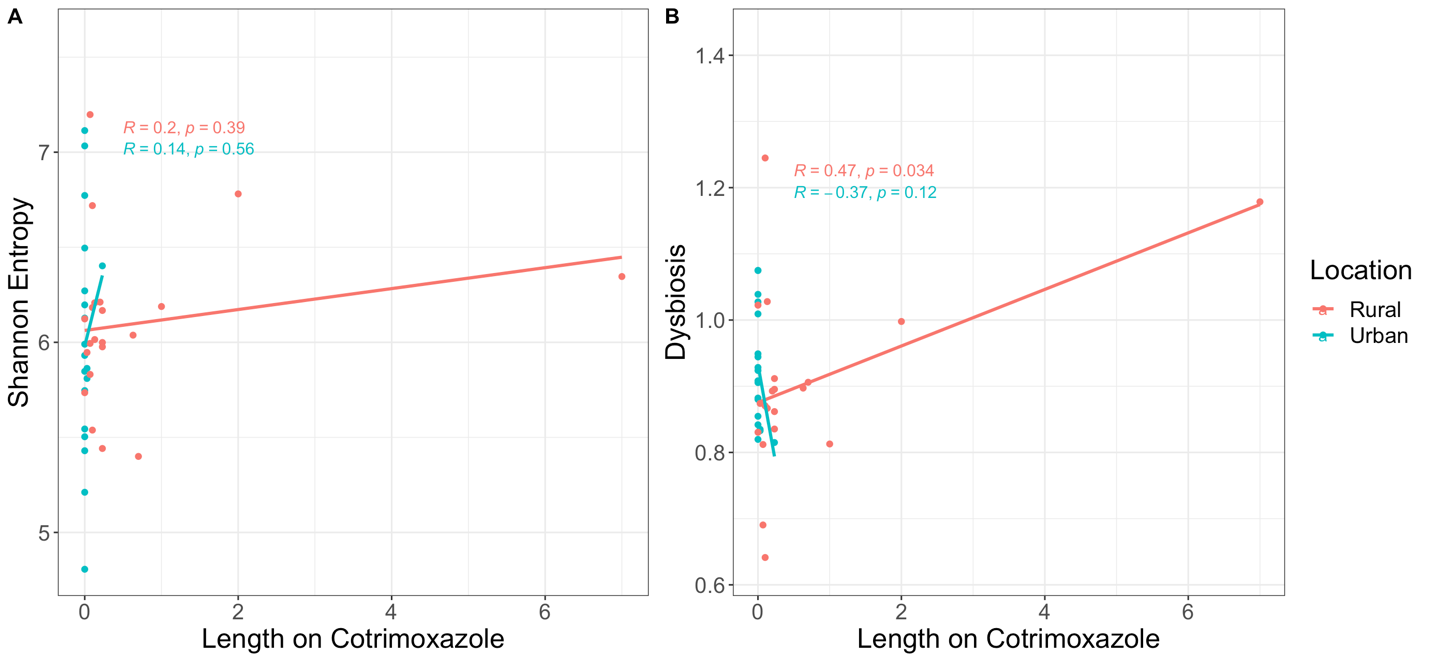


Figure S8: Correlation between the Length of time on Cotrimoxazole (in months) at Baseline (week 0) and alpha diversity (Shannon Entropy) and Dysbiosis (average weighted UniFrac distance of a given sample from each of the health control samples). Values shown for only the ART naïve cohort. A linear regression model was used with an interaction term for rural versus urban location.


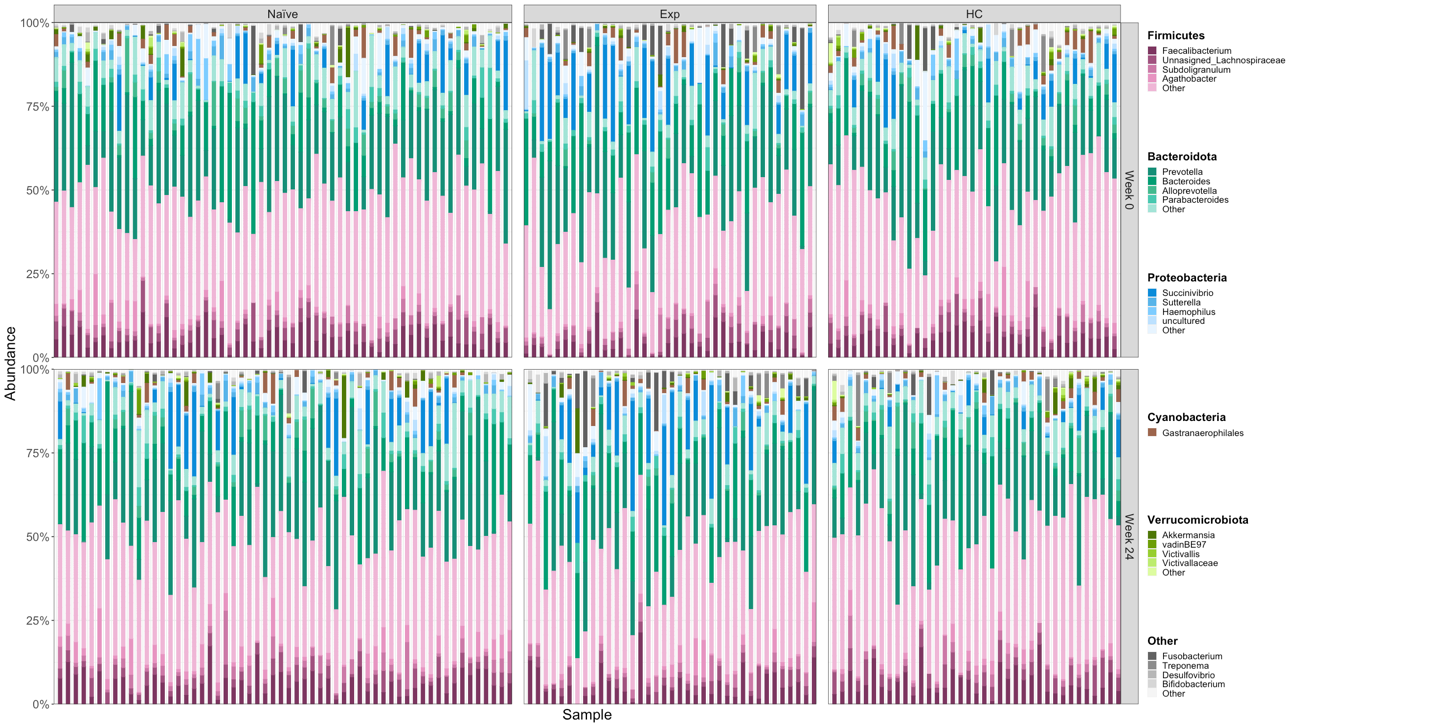


Figure S9: Taxa bar plots. Taxonomic assignments were made using a QIIME2 trained naïve-bayes classifier and the Silva (version 138) taxonomic database [47]. Each color represents a different bacterial genus, and genera within the same Phylum are depicted in different shades of the same color using microshades [101]. Samples are stratified by cohort and time.


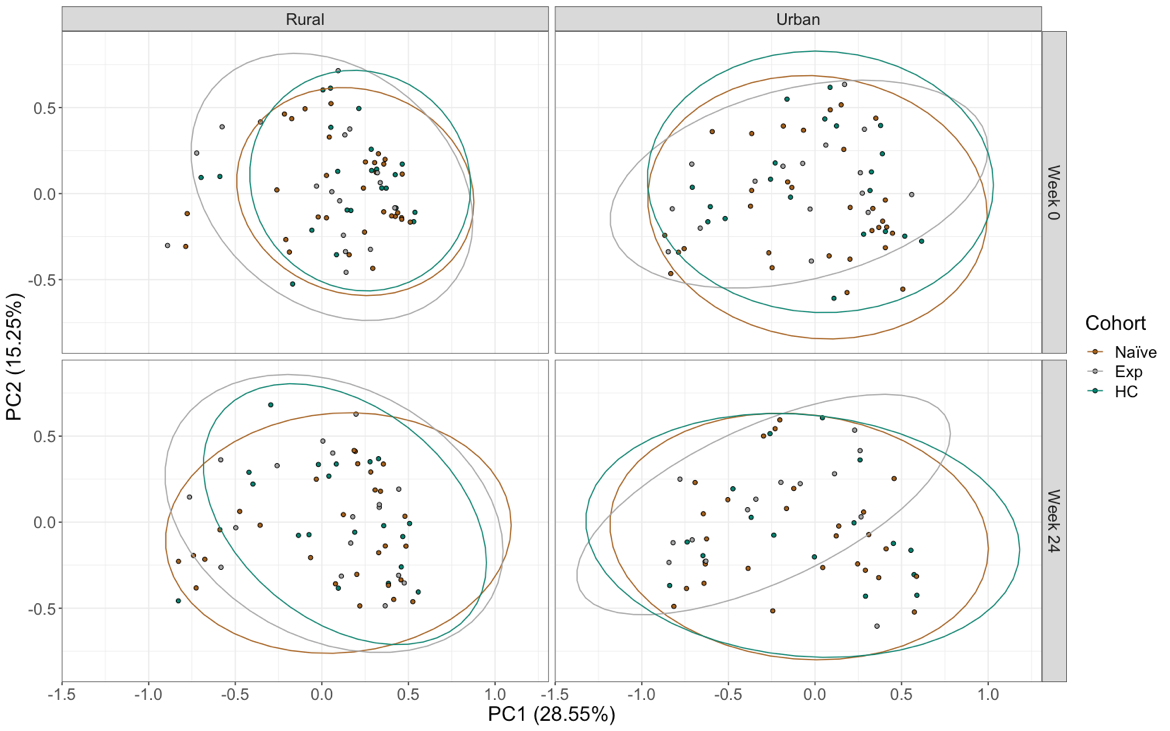


Figure S10: Weighted UniFrac Principal Coordinates Analysis (PCoA). The PCoA was conducted using data from all of the samples, but only a subset of samples are plotted in the different quadrants depending on whether they were from the rural or urban location (columns) or collected at week 0 or week 24 (rows). Points are colored by cohort. Naïve = HIV+ ART Naïve cohort, Exp = HIV+ ART Experienced cohort, HC= Healthy controls. The percent of variation explained by PC1 and PC2 are indicated on the x and y axes respectively.


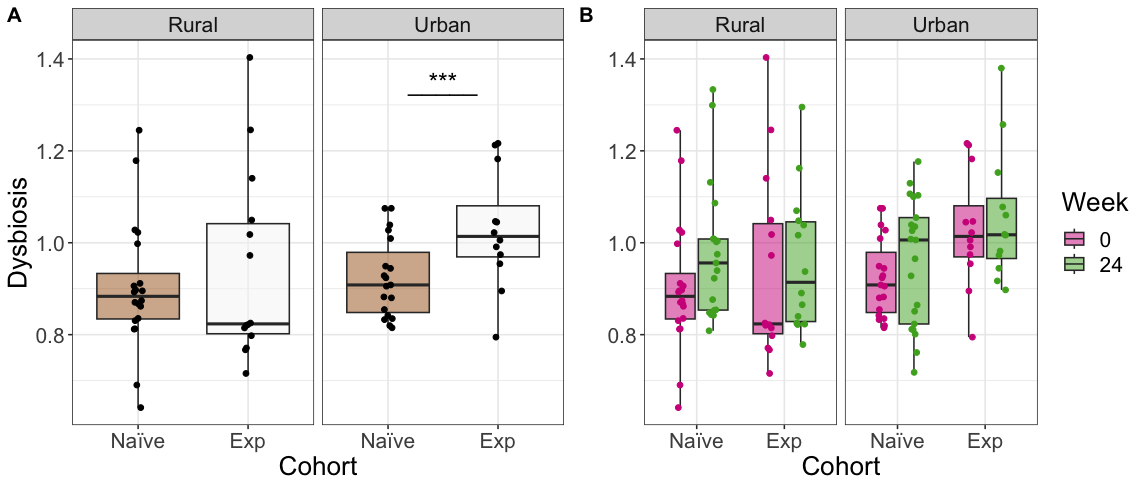
Figure S11: Detailed plots of microbiome dysbiosis levels. A dysbiosis value for each sample was calculated as the average weighted UniFrac distance of that sample from each of the health control samples. Panel A: Inter-cohort comparisons using only baseline (Week 0) values. Statistical significance assessed using a Mann Whitney U test. Panel B: Intra-cohort longitudinal comparisons over time. Statistical significance was assessed using a paired Mann-Whitney U test. P-values are coded as ‘****’ between [0, 0.0001], ‘***’ (0.0001, 0.001], ‘**’ (0.001, 0.01], ‘*’ (0.01, 0.05], with square brackets indicating that the endpoints are included in the interval.

Figure S12: (A) Network summarizing relationships between immune markers (beige nodes) and microbial ASVs (dark pink nodes) at Week 0 for only 2 cohorts: ART experienced and healthy controls. Red edges represent positive associations between an immune marker and microbial feature in the ART Naive cohort. Edge widths are a function of the p-value on the slope of the ART Naive cohort, with thicker edges representing smaller p-values. Relationships were generated by linear models of the form immune marker ~ microbial feature + microbial feature x cohort, with an additional term for read count of the microbial feature. Relationships in this network are limited to those with an FDR-adjusted p-value on the F statistic of the overall regression < 0.2, adjusted R2 > 0.25, p-value on the slope for the Naïve cohort < 0.05 and different from the slope for the Experienced cohort and/or the healthy controls (p<0.05), and maximum absolute value of DFFITS < 2. Names are based on Silva taxonomy assignment for each ASV. Square nodes with more than one listed feature represent highly correlated microbes that were binned using SCNIC. B, C) Scatterplots and fitted regression models of associations between CD4+PD1+ (B), and CD8+PCD103+ (C) immune cells and microbial features. Each circle represents one person, colored by cohort (brown=experienced, green=healthy control). Fitted models for each cohort are shown with colored lines, with dashed lines representing slopes significantly different than 0 (p < 0.05), and dotted lines not significantly different than 0. For all plots, the slope for the experienced cohort is significantly different than zero (pExp, with significance codes for p-values defined as ‘***’ [0, 0.001], ‘**’ (0.001, 0.01], ‘*’ (0.01, 0.05); where square brackets indicate endpoints included in the interval). Adjusted R^2^ (adjR^2^) is provided as a measure of overall model quality.

**
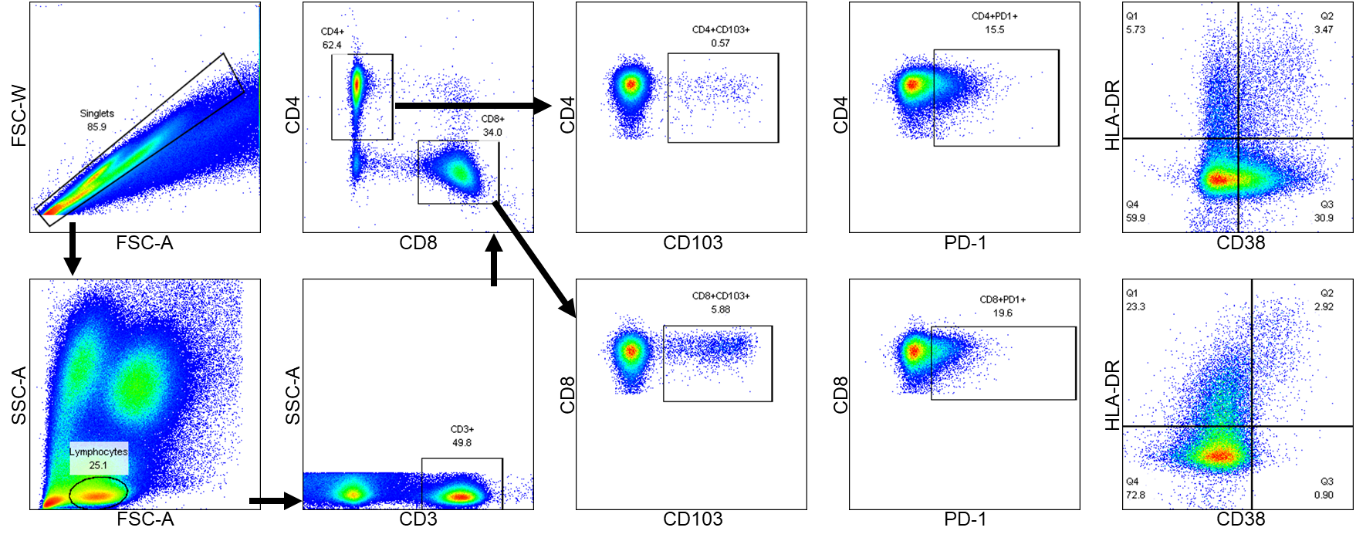
**

Figure S13: Representative staining and gating for CD103, PD1 and T cell activation (CD38+HLA-DR+) on CD4+ and CD8+ T cells in human PBMC. Human PBMC was stained for CD3, CD4, CD8, CD38, CD103, HLA-DR and PD-1 and analyzed by flow cytometry. CD3+ T cells were gated through singlet and lymphocyte gates. CD4+ and CD8+ T cells were then examined for expression of CD103, PD-1 and activation as determined by double positive expression of CD38 and HLA-DR (top right quadrant). Staining of PBMC from a HIV seronegative participant is shown.

Supplemental Tables
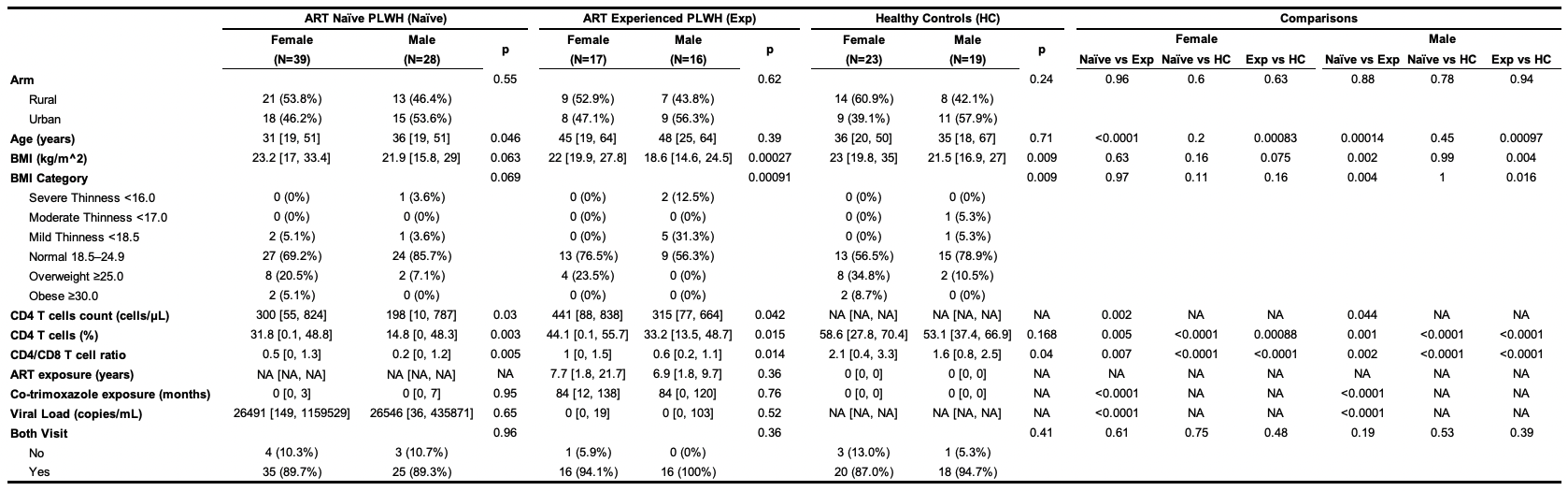


Table S1: Clinical and demographic characteristics of study population by cohort at baseline visit, stratified by sex. P-values were calculated using Mann Whitney U test. NA represents values not collected/ relevant to a particular cohort. Values are reported as the median with the minimum and maximum range indicated in brackets. Virologic failure is defined as PLWH who are on ART but have uncontrolled viral replication(> 200 copies of HIV per milliliter of blood) [24, 25]. BMI categories were determined using World Health Organization (WHO) standards [26].


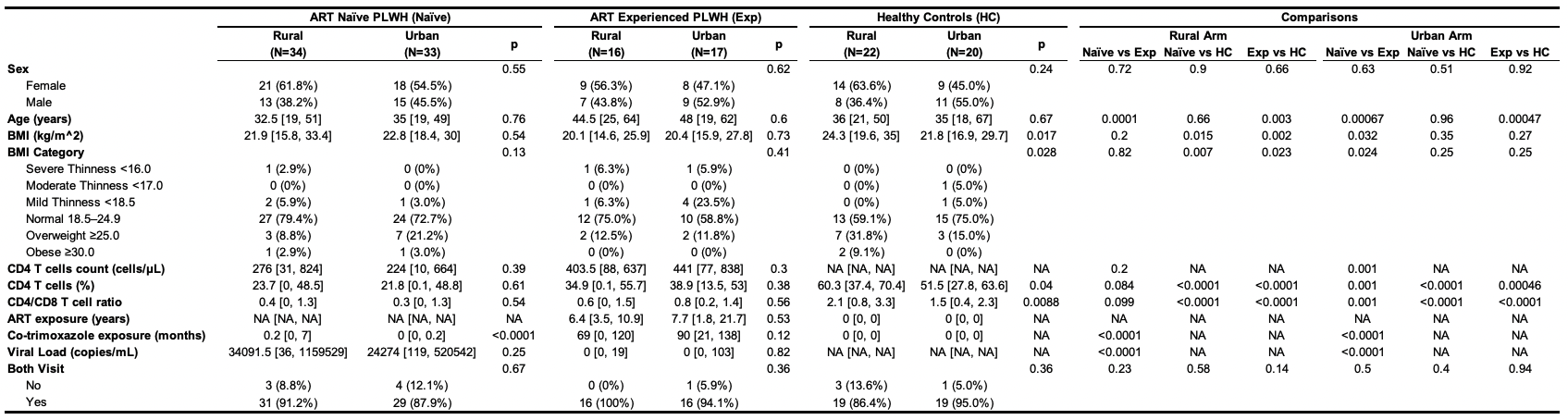


Table S2: Clinical and demographic characteristics of study population by cohort at baseline visit, stratified by the rural versus urban location. P-values were calculated using Mann Whitney U test. NA represents values not collected/ relevant to a particular cohort. Values are reported as the median with the minimum and maximum range indicated in brackets. BMI categories were determined using World Health Organization (WHO) standards [26]


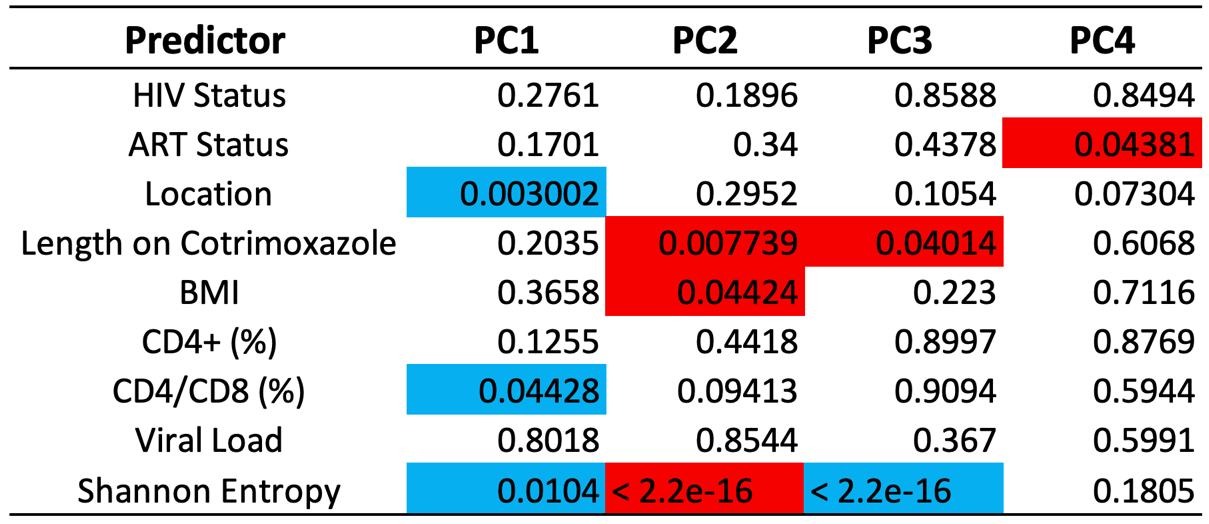


Table S3: Mixed effect linear model p-value results with Weighted UniFrac PCoA arms as response variables. Location decreases from Rural to Urban and ART status increases from no treatment to on treatment. For continuous variables blue indicates negative correlation and red positive correlation.


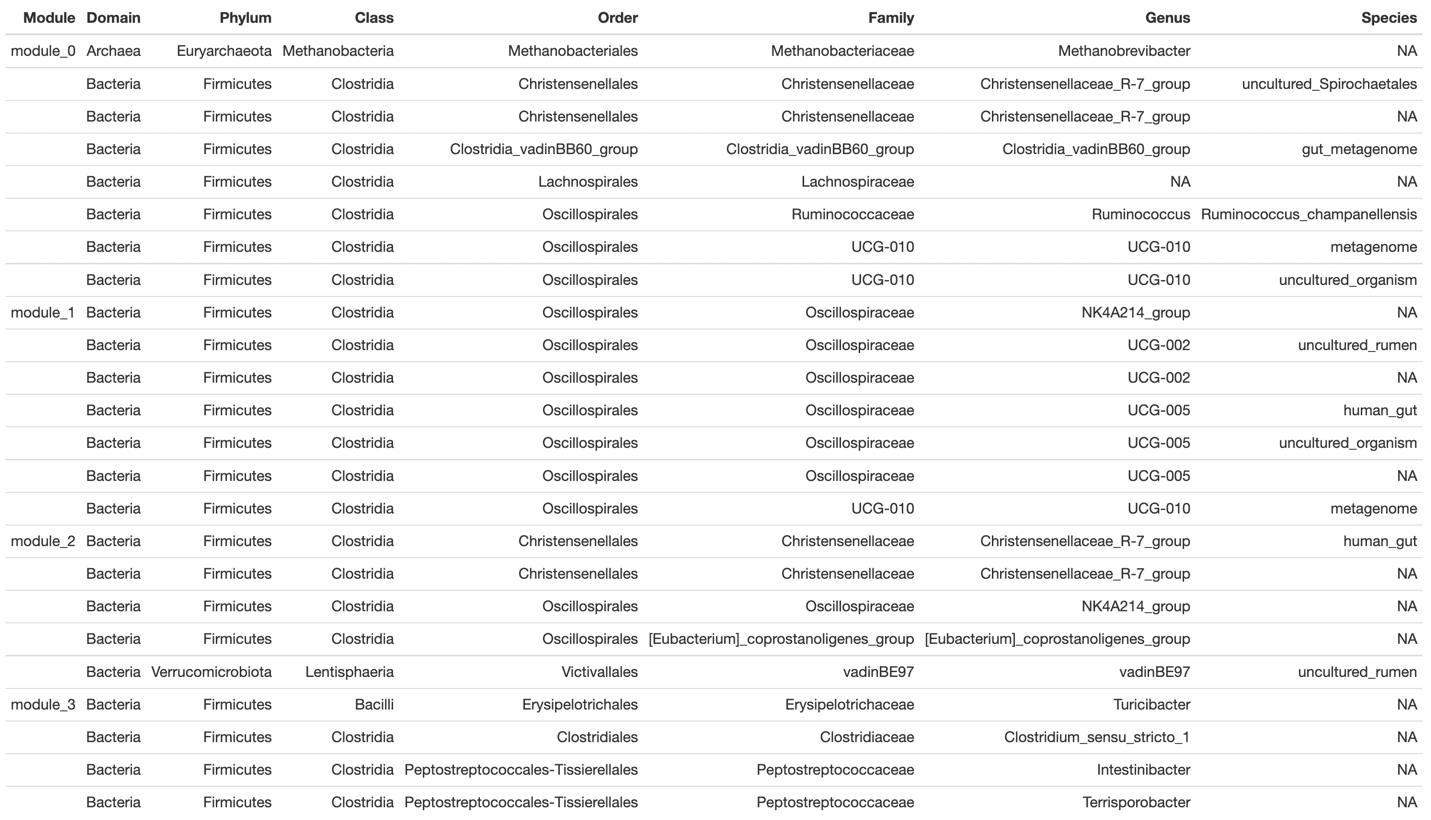


Table S4: Composition of largest modules found using SCNIC. Modules will have over four ASVs assigned to them.
